# Reliability Theory for Measurements with Variable Test Length, Illustrated with ERN and Pe Collected in the Flanker Task

**DOI:** 10.1007/s11336-024-09982-5

**Published:** 2024-07-21

**Authors:** Jules L. Ellis, Klaas Sijtsma, Kristel de Groot, Patrick J. F. Groenen

**Affiliations:** 1 Faculty of Psychology, Open University of the Netherlands, Heerlen, The Netherlands; 2 Tilburg University, Tilburg, The Netherlands; 3 Erasmus University Rotterdam, Rotterdam, The Netherlands

**Keywords:** reliability, event-related potentials, ERN, Pe, Flanker Task, classical test theory

## Abstract

In psychophysiology, an interesting question is how to estimate the reliability of event-related potentials collected by means of the Eriksen Flanker Task or similar tests. A special problem presents itself if the data represent neurological reactions that are associated with some responses (in case of the Flanker Task, responding incorrectly on a trial) but not others (like when providing a correct response), inherently resulting in unequal numbers of observations per subject. The general trend in reliability research here is to use generalizability theory and Bayesian estimation. We show that a new approach based on classical test theory and frequentist estimation can do the job as well and in a simpler way, and even provides additional insight to matters that were unsolved in the generalizability method approach. One of our contributions is the definition of a single, overall reliability coefficient for an entire group of subjects with unequal numbers of observations. Both methods have slightly different objectives. We argue in favor of the classical approach but without rejecting the generalizability approach.

**Supplementary Information:**

The online version contains supplementary material available at 10.1007/s11336-024-09982-5.

## Introduction

This article is based on a consultation request from biological psychologists seeking psychometric advice with respect to reliability issues. They were struggling with the issue of appropriate reliability estimation for the psychophysiological data they collected using a design in which the number of observations per person is a random variable instead of a fixed number, which poses some statistical challenges. Until recently, they relied on methods from classical test theory (CTT), mainly coefficient alpha and the split-half method (e.g., Fabiani et al., 1987) for computing reliability for data characterized by these challenges. A problem with most classical reliability coefficients is that they cannot be applied to these data without discarding large portions of the data (Clayson, 2020). Baldwin et al. (2015) suggested that these simple methods were inappropriate and suggested generalizability theory (GT) as a viable alternative using Bayesian statistics. In this article, we develop two new CTT methods that circumvent this problem using a frequentist approach. Our methods can be applied easily: the first method requires only traditional reliability estimates such as coefficient alpha or $\lambda _{4}$, computed repeatedly, and the second method requires only two observed variances and an observed mean, using 100% of the data. Moreover, our theoretical analysis justifies the computation of an overall reliability coefficient over groups of participants with different numbers of observations. This also leads to a conceptual distinction between ‘reliability’ and ‘test–retest correlation’ even if items are parallel, thus clarifying theoretical issues that were previously unaddressed.

The data relevant to this study are event-related potentials (ERPs) collected during an Eriksen Flanker Task (Eriksen & Eriksen, 1974), but other, albeit similar, data types are also relevant here. A well-known example is the Stroop test (Stroop, 1935). Fabiani et al. (1987) and Hedge et al. (2018) discussed additional stimulus types in the context of CTT reliability estimation. Because we focus on reliability, we do not further discuss other task types for generating similar data sets but concentrate on the Flanker Task data.

In a Flanker Task, participants are repeatedly shown a string of letters (‘SSSSS’, ‘SSHSS’, ‘HHSHH’, ‘HHHHH’) and are instructed to press a button with one hand if the central letter is an ‘H’ and with the other hand if the central letter is an ‘S’. Participants must respond as quickly and as accurately as possible, and although correct responses are observed for the majority of trials, incorrect responses are observed too. On such trials where participants respond incorrectly, specific event-related potentials (ERPs) arise. ERPs are voltage fluctuations in neurons that can be measured from the scalp with the use of electro-encephalography (EEG). Two ERPs that are consistently observed when participants err (in a Flanker Task or in similar experimental designs) are the error-related negativity (ERN, Falkenstein et al., 1991; Gehring et al., 1993) and the error positivity (Pe, Falkenstein et al., 1991). The former peaks between 25 and 100 ms after the commission of an error and is most potent at fronto-central scalp sites. The latter peaks between 200 and 400 ms after the incorrect response and is best observed at centro-parietal locations. Although their precise functional significance is still debated (Olvet & Hajcak, 2008; Overbeek et al., 2005), the ERN is thought to represent early error signaling that is not dependent on the person being aware of having committed the error, and the Pe may represent later, more conscious processing of the error (Nieuwenhuis et al., 2001; O’Connell et al., 2007). The stronger the ERP (i.e., the more negative the ERN and the more positive the Pe), the stronger the neuronal response to committing the error. As the ERN and Pe are only observed when participants err, the number of observations per participant varies, complicating reliability estimation of this data.

Although we developed our reliability theory with ERN and Pe data in mind, it may be applicable to other data where the number of observations is variable. One reviewer noted that “it might be helpful for stimulus-related ERPs, which also tend to have unbalanced trial counts due to artifact rejection,” and we agree with this. Another reviewer pointed out that the situation is similar in cases of agreement coefficients or intraclass coefficients based on multiple raters, if the number of raters or the number of objects is variable, and this is given more attention in Supplementary Material C.

So far, the theoretical psychometric literature has been unaware of the reliability issue that played in this research area. This article extends CTT with new methods for estimating reliability with variable numbers of observations per participant, as is routinely encountered in psychophysiological data such as that of the ERN and Pe. We first describe the Flanker Task, the resulting data matrices, and briefly review reliability methods that have been applied. After stating our assumptions, we present our first method in the form of a theorem and corollary, which deal with potentially non-parallel items. After this, we present our second method, which deals with parallel items. After a computational example and real data examples, we present a theoretical analysis of test–retest correlations, showing that they can generally not be used to estimate reliability. We compare our CTT approach in detail to the GT approaches suggested by Baldwin et al. (2015) and Clayson et al. (2021).

## Flanker Tasks and Resulting Data

### Flanker Tasks

**Fig. 1:**
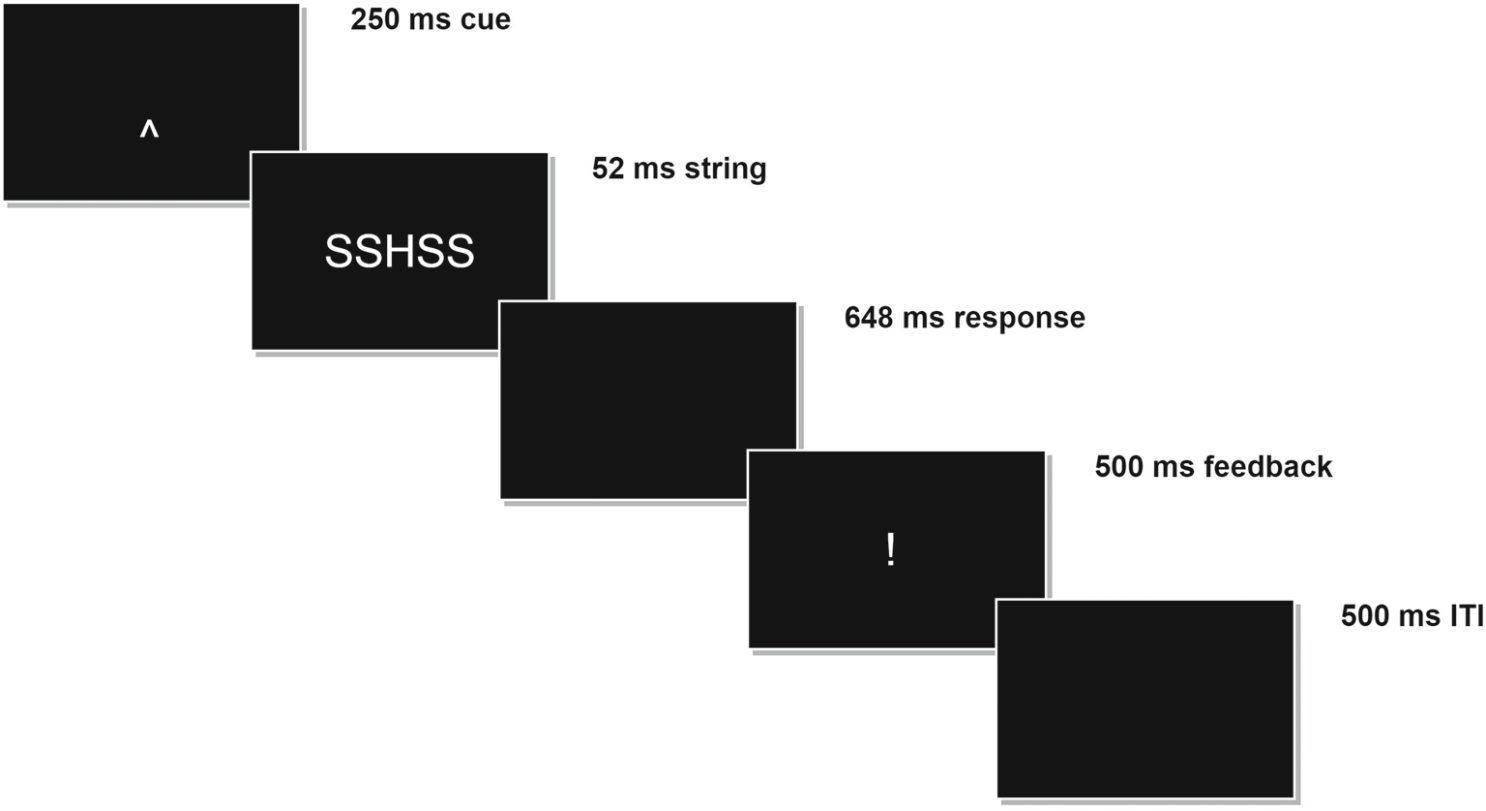
Schematic Representation of a Flanker Task Trial

The Flanker Task used in the present study is a representative version of the Eriksen Flanker Task of which data have already been presented elsewhere (Bernoster et al., 2019; Rietdijk et al., 2014). In this version of the Flanker Task, participants complete 400 trials in which they are shown a letter array of which the central target letter is equal (‘SSSSS’, ‘HHHHH’) or unequal (‘SSHSS’, ‘HHSHH’) to the flanking distractor letters. Participants are instructed to press a predefined button with their right index finger if the central letter (the target) is an ‘H’ and another button with their left index finger if the target is an ‘S’. Trials start with a 250 ms cue (‘⌃’) pointing at the location of the target. Then, the letter array appears for 52 ms, followed by a black screen for 648 ms. During this 700-ms period, participants can respond by pressing one of the buttons. Then, a feedback symbol appears indicating whether their response was correct (‘ooo’), incorrect (‘xxx’), or too late (‘!’). After a 500-ms break (the so-called interstimulus interval or ITI), the next trial starts. The trial sequence is illustrated in Fig. 1. Participants completed 80 trials in a row and had the opportunity to take a break between each series of 80 trials. Within a series, each of the four letter arrays was presented 20 times in a random order to prevent training or fatigue effects from having a systematic effect on certain letter array conditions.

The ERN and Pe data were extracted in line with standard practices in electrophysiological research—the ERN was defined as the mean amplitude at electrode FCz in the 25–100-ms time window, and the Pe was defined as the mean amplitude at electrode Pz in the 200–400-ms time window. Precise information on the recording and (pre-)processing of the data is described in Supplementary Material A.

When given enough time to respond, participants would have each trial correct and no error-related ERPs (i.e., no ERNs or Pe-s) would occur. Therefore, participants respond to trials under time pressure. With adequately chosen presentation, response, and feedback time intervals, time pressure forces participants to make errors. Presenting Flanker Tasks under time pressure is a means for eliciting the data of interest, but because the presence of an ERN / Pe only occurs when the response was incorrect, we will further ignore the correct/incorrect data structure and focus on the trials for which an incorrect answer was given, and thus, an ERN and Pe were elicited.

### Data Matrix

With the Flanker Task, interest resides with psychophysiological activity in response to error. Incorrect responses trigger an ERN and Pe, whereas correct responses do not. There are two ways to represent these data in a data matrix that we will refer to as *spaced* and *condensed*. In the spaced data matrix, each *n*-th column corresponds to the *n*-th trial on which a stimulus was presented. This produces a data matrix containing ERPs when a response was incorrect, interspaced with blanks in other trials. In the condensed data matrix, each *n*-th column corresponds to the *n*-th trial on which the participant made an error. This produces a data matrix with ERPS in consecutive columns at the left side, followed by blanks. A small fictitious example of both data matrices is given in Table 1.

**Table 1:** Small Example of Spaced and Condensed Data Matrix of ERNs

**Spaced**	Trial																	
Participant	1	2	3	4	5	6	7	8	9	10	11	12	13	14	15	16	17	18
1								$-$9				$-$16	$-$10				$-$5	
2								26										
3			$-$3										$-$13					
4	$-$6												$-$6		$-$14			
**Condensed**	Error trial																	
Participant	1	2	3	4	5	6	7	8	9	10	11	12	13	14	15	16	17	18
1	$-$9	$-$16	$-$10	$-$5														
2	26																	
3	$-$3	$-$13																
4	$-$6	$-$6	$-$14															

Because correct responses do not elicit these ERPs, it is disputable whether unavailable ERPs should be considered as missing. The present situation is different from the blanks in a data matrix where, for example, a participant’s age was expected. Because each participant has a particular age, a blank represents a truly missing value that the researcher may wish to track down or treat statistically. This approach does not make sense with alleged ERPs that, in fact, do not exist when responses are correct and therefore are not missing. The blanks in Fig. 1 indicate where positive responses were given, but do not represent missing ERPs. Our reliability method therefore must deal with unequal numbers of scores across participants but not necessarily with missing data. Consequently, we will use the condensed data matrix.

Note that data removed during data cleaning (for ERPs specifically in the artifact rejection step, see Supplementary Material A) are missing even in our definition. This is usually a much smaller part of the data. In our examples with real data, we do not differentiate these missings from the empty cells due to correct responses, but we do not claim that future researchers should necessarily do the same.

We will not differentiate between stimulus types (‘SSSSS’, ‘SSHSS’, ‘HHSHH’, ‘HHHHH’) in the following sections, thus treating them as equivalent, because we want to focus on the methodological innovation. A separate section will discuss how the results of different stimulus types can be integrated.

### Review of Previous Methods to Estimate Reliability

Importantly, as the ERN and Pe represent the neuronal response to committing an error, they do not manifest on trials where a participant responded correctly. It is customary to present the participant with a fixed number of stimuli (e.g., 400) and to compute the participant’s mean ERN and Pe over only the error trials, but it must be noted that the number of error trials is a random variable that can attain different values for different participants. If the mean ERN or Pe of each participant is used as a psychological test score, then the number of error trials corresponds to the concept of test length in reliability theory, but textbooks on CTT do not address the possibility that the test length is a random variable that attains different values for different participants. As a result, it is not directly clear how the reliability of the test scores can be estimated from these data. Klawohn et al. (2020) used the split-half method, and many others used coefficient alpha (Marco-Pallares et al., 2011; Meyer et al., 2013; Olvet & Hajcak, 2009; Pontifex et al., 2010; Rietdijk et al., 2014). A problem with the computation of coefficient alpha in this case is that it requires the same number of observations for each participant. In analyses of ERN and Pe data, this problem is often solved by computing alpha only for a small number of trials, say the first eight trials. A disadvantage of this approach is that it discards all data of participants with fewer than eight trials, as well as data from the ninth trial onward. For example, if this is applied to our ERN data, 69% of the scores are discarded. Other authors (e.g., Clayson et al., 2021) advocated the use of generalizability theory (GT) with multilevel analysis, which does not discard data.

A reason why Clayson et al. (2021) and Clayson and Miller (2017a, b) turned to GT is that it allows a coherent treatment of multiple error sources such as ‘items’ and ‘time.’ Although we agree that this could be a reason to use GT, many studies with ERPs involve only a single error source, such as different trials within the same session, together with maybe fixed factors such as diagnosis group and stimulus type. In these cases, our new CTT methods are simpler and provide additional insights. However, we disagree with the cited authors on one point. Clayson and Miller (2017a, p. 72) state that CTT requires the assumption of parallel items. This has been claimed by authoritative authors on GT too, but we consider this claim misguided (see Sijtsma, 2009a, b; Sijtsma & Pfadt, 2021a, b). In our view, the only fundamental assumption of CTT is that error scores are uncorrelated (Ellis, 2021). In *some* CTT theorems, it is also assumed that items are parallel, but this is not the case for all CTT theorems, and in the absence of parallel items we can still use the part of CTT that does not require parallel items. In order to make this clear, we will precisely state in which formulas we assume parallel items and where we do not.

Both CTT and GT assume uncorrelated error score variables, and for this reason we studied the autocorrelations in the ERN and Pe data of the Flanker Task. This is not the focus of our article, and therefore, this analysis is reported in Supplementary Material B. Our conclusion is that CTT and GT may be applied to these data.

## Reliability for Psychophysiological Data

In this section, we develop a CTT approach to reliability that respects the characteristics of the ERP data collected using the Flanker Task. First, we introduce a CTT definition of reliability for the case that participants do not have the same number of items (here, error trials), typical of Flanker Task ERP data. The reliability defined in this way for the whole group, with varying number of items across participants, is shown to be a weighted average of the reliability estimated within each subgroup with the same number of items. The weights are the subgroup proportions of participants adding up to 1 across all subgroups and are easily derivable from the data, as are the estimates of the other parameters needed. Second, we study the method for parallel items as a special case and derive a result for estimating reliability that is even simpler, because it requires only two observed variances and the harmonic mean of the number of observations per participant. Third, we provide computational examples for estimating reliability for ERP data. Fourth and finally, we study the correlation between test administrations that have item-by-item parallelism between administrations but not within the same administration. We show that in this case the test scores would not be parallel, and therefore, there is no reason to expect that the correlation of the two test scores is equal to their reliability. We also show that if the items *within the test administrations* are parallel, then the situation simplifies considerably and the reliability can be estimated from the correlation between two administrations if the harmonic means of the test lengths are equal.

### Reliability if the Number of Items is a Random Variable

#### Assumptions

Let $$X_{1},X_{2},\ldots $$ be an infinite sequence of observable score variables, where $$X_{i}$$ is the observable score variable on trial *i*. The variables are called “observable” because we assume that not all of them are observed for all participants. In this study, this means that a variable is observed if an ERN score and a Pe score are observed and recorded for a participant. We will also say that, following psychometrics jargon, each variable $$X_{i}$$ is an *item score variable*, or even shorter, an *item; *the *i*-th column in the condensed data matrix is a sample of $$X_{i}$$. Let *N* be the number of observed trials; *N* is a random variable. We assume that the variables that are observed are $$X_{1},X_{2},\ldots ,X_{N}$$, where *N* can have different values for different participants. We assume $$N\ge 1$$ for all participants. In practical situations, *N* would also be bounded from above by some fixed number *m* (in our study, $$m=400)$$, but there is no mathematical need to assume that here.

We assume CTT for the observable variables: For each $i\in \mathbb {N}$, there are variables $T_{i}$ and $E_{i}$ such that for all $i,j,k\in \mathbb {N}$ with $k\ne i$,

A1 $$\begin{aligned}{} & {} {X_i} = {T_i} + {E_i} \end{aligned}$$

A2 $$\begin{aligned}{} & {} \text {Cov}\!\left( {{E_i},{T_j}} \right) = 0 \end{aligned}$$

A3 $$\begin{aligned}{} & {} \text {Cov}\!\left( {{E_i},{E_k}} \right) = 0 \end{aligned}$$

Assumption A3 refers to uncorrelated errors. We need it in some derivations but not in all. We further assume that the expected measurement error does not depend on the number of observations; that is, for all $i,n\in \mathbb {N}$,

A4 $$\begin{aligned} \mathbb {E}(E_{i}|N = n) = 0 \end{aligned}$$

We further assume that the true scores and error scores are still uncorrelated if one considers only a subpopulation with a fixed number of observations: for all $i,j,n\in \mathbb {N}$,

A5 $$\begin{aligned} {\textrm{Cov}}({E}_{i}, T_{j}|N = {n}) = 0 \end{aligned}$$

Finally, we assume that the variables $N,X_{i}$, $T_{i}$ and $E_{i}$ have finite second moments, both unconditionally and conditionally on *N*.

#### Variance Decomposition of Total Scores

Since participants differ in their number of observations, it is convenient to define each participant’s overall test score not as the raw sum score, but rather as the mean of available item scores of the participant. We therefore define the test (or total) observed score, the test (or total) true score, and the test (or total) error score as

$$\begin{aligned} X_{+}:= & {} \sum \limits _{i=1}^N X_{i} /N,\\ T_{+}:= & {} \sum \limits _{i=1}^N T_{i} /N,\\ E_{+}:= & {} \sum \limits _{i=1}^N E_{i} /N. \end{aligned}$$

Then $X_{+}=T_{+}+E_{+}$ but, now that the number of summands is variable, it is not obvious whether at the group level we have that $\textrm{Var}\left( X_{+} \right) =\textrm{Var}\left( T_{+} \right) +\textrm{Var}\left( E_{+} \right)$. This is what we prove next.

##### Lemma 1

Assume A1, A2, A4 and A5. Then

$$\begin{aligned} \textrm{Cov}\!\left( E_{+},T_{+} \right) =0. \end{aligned}$$

##### Proof

By the law of total covariance, we have

$$\begin{aligned} \textrm{Cov}\!\left( E_{+},T_{+} \right) =\mathbb {E}(\textrm{Cov}\left( E_{+},T_{+}\vert N \right) )+\textrm{Cov}(\mathbb {E}(E_{+}\vert N),\mathbb {E}(T_{+}\vert N)). \end{aligned}$$

In the first term on the right, using the property that $$\textrm{Cov}\!\left( aY,bZ \right) =ab\,\textrm{Cov}(Y,Z)$$ if *Y* and *Z* are random variables and *a* and *b* are scalars (here, we use $$a=b=n^{-1})$$, and assumption A5, we obtain

$$\begin{aligned} \textrm{Cov}\!\left( E_{+},T_{+}\vert N=n \right) = \sum \limits _{i=1}^n \sum \limits _{j=1}^n {n^{-2}\textrm{Cov}\left( E_{i},T_{j}\vert N=n \right) } =0. \end{aligned}$$

Therefore, we conclude that $\mathbb {E}\left( \textrm{Cov}\left( E_{+},T_{+}\vert N \right) \right) =0.$ In the second term on the right, we have

$$\begin{aligned} \mathbb {E}\left( E_{+} \vert {N=n}\right) =\mathbb {E}\left( {\sum \limits _{i=1}^N E_{i} /n} \vert {N=n}\right) =\sum \limits _{i=1}^n {n^{-1}\mathbb {E}\left( E_{i} \vert {N=n}\right) } =0. \end{aligned}$$

Therefore, $$\textrm{Cov}\!\left( \mathbb {E}\left( E_{+} \vert \,N\right) ,\mathbb {E}\left( T_{+} \vert \,N\right) \right) =0$$. $$\square $$

From Lemma 1, it follows immediately that $\textrm{Var}\left( X_{+} \right) =\textrm{Var}\!\left( T_{+} \right) +\textrm{Var}\!\left( E_{+} \right)$.

#### Conditional and Unconditional Reliability

We define reliability generically as the ratio of true score variance to observed score variance. This is consistent with the definitions of many previous authors in CTT (e.g., Cho, 2021; Guttman, 1953; Novick, 1966; Raykov & Marcoulides, 2017). Define the *unconditional* reliability of the test observed score as

$$\begin{aligned} \textrm{Rel}\left( X_{+} \right) :=\frac{\textrm{Var}(T_{+})}{\textrm{Var}(X_{+})}. \end{aligned}$$

We set $\textrm{Rel}\left( X_{+} \right) :=0$ if $\textrm{Var}\!\left( X_{+} \right) =0.$ We will now stratify the participant population based on *N* and then consider some parameters defined on the stratification. First, we assume that we can estimate the reliability of the test observed score in the subpopulation where the number of observations equals *n*. This is the *conditional* reliability of the test observed score, defined as

$$\begin{aligned} \rho _{n}:=\frac{\textrm{Var}(T_{+}\vert N=n)}{\textrm{Var}(X_{+}\vert N=n)}. \end{aligned}$$

We set $\rho _{n}:=0$ if $\textrm{Var}\left( X_{+} \vert {N=n}\right) =0$, so that $\textrm{Var}\left( T_{+} \vert {N=n}\right) =\rho _{n}\textrm{Var}(X_{+}\vert N=n)$ in all cases. Furthermore, we write the conditional observed variance as

$$\begin{aligned} \sigma _{n}^{2}:=\textrm{Var}(X_{+}\vert N=n) \end{aligned}$$

and the fraction of the subjects with *n* observations as

$$\begin{aligned} \pi _{n}:=P(N=n). \end{aligned}$$

If the number of observed trials is bounded by some $m\in \mathbb {N}$, then we can simply write $\pi _{n}=0$ for $n>m$. We express the unconditional reliability, $\textrm{Rel}\left( X_{+} \right)$ in terms of the conditional reliabilities, $\rho _{n}$. Note that the following result does not require uncorrelated errors.

##### Theorem 1

Assume A1, A2, A4 and A5. The *unconditional* reliability of the total observed score $X_{+}$ is then given by

$$\begin{aligned} \textrm{Rel}\left( X_{+} \right) =1-\frac{{\sum }_{n=1}^\infty {(1-\rho _{n})\sigma _{n}^{2}\pi _{n}} }{\textrm{Var}(X_{+})}. \end{aligned}$$

##### Proof

By the law of total variance, we have

$$\begin{aligned} \textrm{Var}\left( X_{+} \right) =\mathbb {E}\left( \textrm{Var}\left( X_{+} \vert \,N\right) \right) +\textrm{Var}(\mathbb {E}\left( X_{+} \vert \,N\right) ) \end{aligned}$$

and

$$\begin{aligned} \textrm{Var}\left( T_{+} \right) =\mathbb {E}\left( \textrm{Var}\left( T_{+} \vert \,N\right) \right) +\textrm{Var}(\mathbb {E}\left( T_{+} \vert \,N\right) ). \end{aligned}$$

Assumption A4 implies $\mathbb {E}\left( E_{i} \vert \,N\right) =0$. Combining this result with the CTT definition $X_{+}=T_{+}+E_{+}$ and its expectation for subgroups, $\mathbb {E}{(X}_{+}\vert N)=\mathbb {E}(T_{+}\vert N)+\mathbb {E}(E_{+}\vert N)$, we have $\mathbb {E}\left( X_{+} \vert \,N\right) =\,\mathbb {E}\left( T_{+} \vert \,N\right)$; hence, the variance terms on the right of the two former equations vanish if we subtract $\textrm{Var}\left( T_{+} \right)$ from $\textrm{Var}\left( X_{+} \right)$ to obtain $\textrm{Var}\left( E_{+} \right)$. Therefore,

$$\begin{aligned} \textrm{Var}\left( E_{+} \right)= & {} \textrm{Var}\left( X_{+} \right) -\textrm{Var}\left( T_{+} \right) \\= & {} \mathbb {E}\left( \textrm{Var}\left( X_{+} \vert \,N\right) \right) -\mathbb {E}\left( \textrm{Var}\left( T_{+} \vert \,N\right) \right) \\= & {} \sum \limits _{n=1}^\infty {\textrm{Var}(X_{+}\vert N=n)\times } \pi _{n}-\sum \limits _{n=1}^\infty {\textrm{Var}(T_{+}\vert N=n)} \times \pi _{n} \end{aligned}$$

Since $\textrm{Var}\left( T_{+} \vert \,{N=n}\right) =\rho _{n}\sigma _{n}^{2}$, we have

$$\begin{aligned} \textrm{Var}\left( E_{+} \right)= & {} \sum \limits _{n=1}^\infty {\sigma _{n}^{2}\pi _{n}} -\sum \limits _{n=1}^\infty {\rho _{n}\sigma _{n}^{2}\pi _{n}}\\= & {} \sum \limits _{n=1}^\infty {(1-\rho _{n})\sigma _{n}^{2}\pi _{n}}.\qquad \qquad \qquad \qquad \qquad \qquad \qquad \qquad \qquad \qquad \end{aligned}$$

$$\square $$

The key principle of Theorem 1 is that, although we should not average the reliability coefficients from different groups, we may average the error variances if the mean error is 0 in each group. This provides a simple estimation method for reliability when different participants have responded to different numbers of items, based on general assumptions.

As an aside, one may note the resemblance of the formula in the theorem with the formula underlying stratified alpha. Suppose a test consists of *G* subtests, each measuring a different aspect of an overarching attribute of greater complexity than each of the aspects it represents, such as intelligence. Let $\sigma _{g}^{2}$ denote the variance of the score on subtest *g*; $\rho _{g}$ the reliability of subtest *g*; and $\sigma _{X}^{2}$ the variance of the total sum score across the *G* subtests; then, the reliability of the total score equals (Lord & Novick, 1968, exercise 4.5; Nunnally, 1978, p. 248)

$$\begin{aligned} \textrm{Rel}\left( X_{+} \right) =1-\frac{{\sum }_{g=1}^G {(1-\rho _{g})\sigma _{g}^{2}} }{\textrm{Var} (X_{+})}. \end{aligned}$$

This is called a stratified reliability coefficient, and it is called stratified alpha if $\rho _{g}$ is replaced by the corresponding coefficient $\alpha _{g}$ of subtest *g*; other reliability coefficients can be stratified similarly (Ogasawara, 2009). The stratification of the treatment of the Flanker data concerns the participant population rather than the item set; therefore, both equations are applicable in different situations.

Theorem 1 uses the ‘true’ population values of the conditional reliabilities $\rho _{n}$, but these are usually not known exactly. Any estimation method that produces correct reliability estimates can be used here. We will now describe how, moreover, coefficient alpha can be used to obtain a lower bound to the unconditional reliability. Let $\alpha _{n}$ be the value of coefficient alpha (Cronbach, 1951; Novick & Lewis, 1967; Ten Berge & Sočan, 2004; Sijtsma & Van der Ark, 2020) in the subpopulation with $N=n$. Assuming uncorrelated errors (assumption A3), a standard result is that $\alpha _{n}\le \rho _{n}$, irrespective of the population or any selection thereof. Substitution of $\alpha _{n}$ for $\rho _{n}$ in Theorem 1 yields the following result.

##### Corollary 1

Assume A1, A2, A3, A4 and A5. Then

$$\begin{aligned} \textrm{Rel}\left( X_{+} \right) \ge 1-\frac{{\sum }_{n=1}^\infty {\left( 1-\alpha _{n} \right) \sigma _{n}^{2}\pi _{n}} }{\textrm{Var}\left( X_{+} \right) }. \end{aligned}$$

The proof follows immediately from $\alpha _{n}\le \rho _{n}$. The quantity at the right hand may be named *length-stratified alpha*. Based on Corollary 1, we suggest to estimate coefficient alpha in each subgroup with a fixed number of observations, use them as lower bounds of the conditional reliabilities, and aggregate them into the length-stratified alpha, which may then serve as a lower bound of the unconditional reliability of the total score. The old method of using alpha in this situation was to pick a minimum number of available trials, say $m=12$, and then compute coefficient alpha with *m* items, thus discarding the available item scores $X_{i}$ with $i>m$ and discarding the participants with $N<m$. For the ensuing coefficient alpha, it would, however, be unclear whether it is greater than, less than, or equal to the unconditional reliability. Our length-stratified alpha has the advantage that all data and all participants are used in the estimation and that the direction of the bias is clear: it yields a lower bound to the unconditional reliability.

Although alpha has been heavily criticized, we have the considered opinion that it is appropriate in the present situation. Only if the data are highly multidimensional will coefficient alpha show a large theoretical discrepancy with respect to the true reliability, but otherwise it closely approximates reliability from below (Sijtsma & Pfadt, 2021a). However, if one wants to avoid coefficient alpha, it may be replaced in Corollary 1 by any other lower bound or lower-bound estimate of reliability, such as Guttman’s $\lambda _{2}$ (Guttman, 1945)

#### Simple Formula for Parallel Items

Let us now assume furthermore that the items are parallel. This means they satisfy assumptions A1, A2 and A3, and for all $i,j\in \mathbb {N}$

A6 $$\begin{aligned} T_{i}= & {} T_{j}, \end{aligned}$$

A7 $$\begin{aligned} {\textrm{Var}}({E}_{i})= & {} {\textrm{Var}}(E_{j}). \end{aligned}$$

These assumptions imply that the items have equal variances and equal correlations. For simplicity, denote $\varepsilon ^{2}:=\textrm{Var}\left( E_{i} \right)$, $\tau ^{2}:=\textrm{Var}\left( T_{i} \right)$, $\sigma ^{2}:=\textrm{Var}\left( X_{i} \right)$, and $\rho =\textrm{Cor}\left( X_{i},\,X_{j} \right)$. We assume the latter correlation is defined, hence $\sigma ^{2}>0$, and then, standard CTT results are that $\tau ^{2}=\,\rho \sigma ^{2}$ and $\varepsilon ^{2}=(1-\rho )\sigma ^{2}$. We furthermore assume that the error variances and covariances are independent of the number of items; that is, for all $i,j\in \mathbb {N}$, with $i\ne j$,

A7a $$\begin{aligned} \textrm{Var}(E_{i}\vert N)=\textrm{Var}(E_{i})\,\textrm{and}\, \textrm{Cov}(E_{i},\,E_{j}\vert N)=0. \end{aligned}$$

This means that responses of subjects with longer tests are not more or less reliable than responses of other subjects, and errors remain uncorrelated within groups of the same test length.

##### Theorem 2

Assume A1, A2, A3, A4, A5, A6, A7 and A7a and $\textrm{Var}\left( X_{+} \right) >0$. Then

$$\begin{aligned} \textrm{Rel}\left( X_{+} \right)= & {} 1-\frac{\mathbb {E}\left( N^{-1} \right) (1-\rho )\sigma ^{2}}{\textrm{Var}(X_{+})}\\= & {} \frac{\rho }{\rho +\mathbb {E}\left( N^{-1} \right) (1-\rho )\mathrm {\,}}\\= & {} \frac{1}{1-\mathbb {E}\left( N^{-1} \right) }-\frac{\mathbb {E}\left( N^{-1} \right) }{1-\mathbb {E}\left( N^{-1} \right) }\frac{\sigma ^{2}}{\textrm{Var}(X_{+})}. \end{aligned}$$

##### Proof

By the law of total variance, and noting that $\mathbb {E}\left( E_{+} \vert \,N\right) =0$ (first step below) and, because due to A7 and A7a, which is true irrespective of the number of trials, *N*, we can write $\textrm{Var}\left( E_{+} \vert \,N\right) =\textrm{Var}\left( N^{-1}\sum E_{i} \vert N \right) =N^{-2}.N.\textrm{Var}\left( E_{i} \vert \,N\right) =N^{-1}\textrm{Var}\, (E_{i})=N^{-1}\varepsilon ^{2}$ (second step below), whereas $\textrm{Var}\left( X_{+} \right) >0$ implies $\sigma ^{2}>0$ so that $\varepsilon ^{2}=(1-\rho )\sigma ^{2}$ (fourth step below); we can readily derive

$$\begin{aligned} \textrm{Var}\left( E_{+} \right)= & {} \mathbb {E}\left( \textrm{Var}\left( E_{+} \vert \,N\right) \right) +\textrm{Var}\left( \mathbb {E}\left( E_{+} \vert \,N\right) \right) \\= & {} \mathbb {E}\left( \textrm{Var}\left( E_{+} \vert \,N\right) \right) \\= & {} \mathbb {E}\left( N^{-1}\varepsilon ^{2} \right) \\= & {} \mathbb {E}\left( N^{-1} \right) \varepsilon ^{2}\\= & {} \mathbb {E}\left( N^{-1} \right) (1-\rho )\sigma ^{2}. \end{aligned}$$

This yields the first equation in Theorem 2. To obtain the second equation, we take the next steps. Because all item true scores are parallel, it follows that $T_{+}$, which is the mean of the item true scores, equals $T_{+}=T_{i}$; hence, $\textrm{Var}\left( T_{+} \right) =\tau ^{2}=\rho \sigma ^{2}$, and $\textrm{Var}\left( X_{+} \right) =\textrm{Var}\left( T_{+} \right) +\textrm{Var}\left( E_{+} \right) =\rho \sigma ^{2}+\mathbb {E}\left( N^{-1} \right) (1-\rho )\sigma ^{2}$. This yields the second equation in Theorem 2. To obtain the third and final equation, we notice that $\textrm{Var}\left( X_{+} \right) /\sigma ^{2}=\rho +\mathbb {E}\left( N^{-1} \right) (1-\rho )$, and solving for $\rho$ yields

$$\begin{aligned} \rho =\frac{1}{1-\mathbb {E}(N^{-1})}\times \frac{\textrm{Var}(X_{+})}{\sigma ^{2}}-\frac{\mathbb {E}(N^{-1})}{1-\mathbb {E}(N^{-1})}. \end{aligned}$$

Multiplying both sides with $\frac{\sigma ^{2}}{\textrm{Var}(X_{+})}$ yields on the left-hand side

$$\begin{aligned} \frac{\rho \sigma ^{2}}{\textrm{Var}(X_{+})}=\frac{\textrm{Var}(T_{+})}{\textrm{Var}(X_{+})}=\textrm{Rel}(X_{+}), \end{aligned}$$

so that we obtain

$$\begin{aligned} \textrm{Rel}\left( X_{+} \right) =\frac{1}{1-\mathbb {E}(N^{-1})}-\frac{\mathbb {E}\left( N^{-1} \right) }{1-\mathbb {E}\left( N^{-1} \right) }\frac{\sigma ^{2}}{\textrm{Var}(X_{+})}. \end{aligned}$$

This is the third equation in Theorem 2. $\square$

##### Corollary 2

Under the circumstances of Theorem 2,

- A sample estimate of the unconditional reliability can be computed from estimates of $\mathbb {E}\left( N^{-1} \right)$, $\sigma ^{2}$, and $\textrm{Var}(X_{+})$.
- If we write the harmonic mean of *N* as $H=1/\,\mathbb {E}\left( N^{-1} \right)$, then
  $$\begin{aligned} \textrm{Rel}\left( X_{+} \right) =\frac{H\rho }{1+(H-1)\rho }. \end{aligned}$$

The second part of Corollary 2 says that we can generalize the Spearman–Brown formula to a situation with variable test lengths by substituting the harmonic mean of the test lengths.

As a generalization of the results obtained thus far, we mention the possibility to include subgroupings of the population replacing subgroupings based on the number of items that elicited ERPs or combining the two subgrouping variables. Reliability results are largely like the results obtained thus far. Definitions and proofs are provided in Supplementary Material C.

#### Examples

**Example of Theorem** 1. This example uses the ERN data obtained from 158 participants. We consider a total of 400 letter series presentations, making no distinction between the four different letter series (100 presentations each). Together, these participants realized 50 different values of the number of trials, $N=n$, running from 0 trials (8 participants, the highest frequency for any of the 50 values of *n*) through 122 trials (1 participant, the lowest frequency also realized with 15 different values of *n*). Participants with 0 (8 participants) or 1 (7 participants) realized trials are not useful because the conditional alpha is undefined in these groups. Groups defined by $N=n$ with one participant cannot be used either because the conditional sample variance is undefined in such groups. Note that with the method used in the proof of the lemma, the results still hold if stratification is done with groups that combine groups of the form $[N=n]$. Therefore, we used deciles of the subjects with $N\ge 2$; see Table 2. In each group, there is a range in the number of available trials. For example, in the first decile *N* ranged from 2 to 4, and in the second decile *N* ranged from 5 to 7. The conditional alphas were computed with only the trial numbers on which all participants in the group had a score, that is, the minimum number of trials in that group. For example, in the first decile group, $\alpha _{n}$ was computed with two items, even though some subjects had four items, and in the second decile group, $\alpha _{n}$ was computed with five items, even though some participants had seven items. In total, 2509 out of 3011 observations were used (83%), and this percentage can grow to 100% if more participants are added to the sample, such that each group of the form $[N=n]$ is large enough to estimate $\alpha _{n}$ without combining groups. In contrast, only 31% of the observations would be used if a single coefficient alpha is computed for the first eight items, and 42% would be used if alpha is computed with the number of items that utilizes the largest percentage of the data, which is 18 items. The table further lists the estimates of $\pi _{n}$, $\alpha _{n}$, $\sigma _{n}^{2}$, and $\left( 1-\alpha _{n} \right) \sigma _{n}^{2}\pi _{n}$ per decile group. The sum of the estimates of $\left( 1-\alpha _{n} \right) \sigma _{n}^{2}\pi _{n}$ is 15.986, and the sample variance of $X_{+}$ in the entire group is 47.390. Therefore, the estimated value of length-stratified alpha is

$$\begin{aligned} 1-\frac{{\sum }_{n=1}^\infty {\left( 1-\alpha _{n} \right) \sigma _{n}^{2}\pi _{n}} }{\textrm{Var}\left( X_{+} \right) }\approx 1-\frac{\mathrm {15.986}}{\mathrm {47.390}}=0.663. \end{aligned}$$

It should be noted that each group has fewer than 20 participants and that each $\alpha _{n}$ may have a large standard error. Nevertheless, the total estimate of length-stratified alpha might have an acceptable standard error, because it is based on a weighted average of the $\alpha _{n}$s. For example, simulation of 1000 samples with parallel items with reliability 0.1887 each, using the test lengths in the column “Number of Items Used” of Table 2, with 13 participants per group, showed that a mean length-stratified alpha of 0.656 had a bias of only $-0.007$ (compared to the outcome 0.663 in a single simulation with subgroups of ${10}^{6}$ participants) and a standard error of 0.074. In comparison, with the same parameters, a single sample of 130 subjects with 9 items would have an alpha with standard error 0.042. Thus, stratification increased the standard error—as usual—but the effect may be modest. An extensive study of the standard error of length-stratified alpha would be interesting but is beyond the scope of this article.

**Table 2:** Statistics For The Computation of Length-Stratified Alpha

Decile of *N*	Number of Participants	Number of Items Used	$\pi _{n}$	$\alpha _{n}$	$\sigma _{n}^{2}$	$\left( 1-\alpha _{n} \right) \sigma _{n}^{2}\pi _{n}$
1	15	2	0.105	0.508	121.013	6.242
2	11	5	0.077	0.700	77.562	1.789
3	18	8	0.126	0.527	40.547	2.414
4	15	11	0.105	0.676	39.855	1.353
5	13	15	0.091	0.572	26.489	1.031
6	15	18	0.105	0.880	73.058	0.922
7	12	21	0.084	0.753	26.548	0.551
8	16	25	0.112	0.692	23.156	0.799
9	15	31	0.105	0.583	13.355	0.584
10	13	41	0.091	0.920	41.317	0.301
Total	143				47.390	15.986

Note. $N =$ number of items; $\pi _{n} =$ number of participants in decile / total number of participants (143); $\alpha _{n} =$ coefficient alpha in decile; $\sigma _{n}^{2} =$ variance of sum score in decile group

**Computational Example of Theorem** 2. We start with a computational example, using only a small subsample of persons to clarify the steps needed. Table 3 shows the ERN scores of seven participants and the variables $X_{+}$, *N*, and 1/*N* derived from the data. The sample variance of all the raw ERP scores in Table 3 ($-$7.32 to $-$11.73, spanning six columns) is 127.0444, which we use as an estimate of $\sigma ^{2}$; the sample variance of $X_{+}$ is 52.7442, and the sample mean of 1/*N* is 0.2357. If we substitute this in the last equation of Theorem 2, thus assuming parallel trials, we obtain

$$\begin{aligned} \textrm{Rel}\left( X_{+} \right) \approx \frac{1}{1-\mathrm {\,0.2357}}-\frac{\mathrm {\,0.2357}}{1-\mathrm {\,0.2357}} \frac{\mathrm {127.0444}}{\mathrm {52.7442}}=0.566. \end{aligned}$$

Using the sample variance of subjects with different *N* as an estimate of $\sigma ^{2}$ is justified because each column is assumed to have the same expectation and variance, as we assume parallel items and scores independent of *N*.

**Table 3:** Computational Example for Seven Participants (out of 143)

Person	ERN Scores						$X_{+}$	*N*	1/*N*
P01	$-$7.32	1.44	3.78	3.87			0.4425	4	0.2500
P02	$-$3.85	$-$9.87					$-$6.8600	2	0.5000
P03	2.61	$-$12.34	12.95	$-$10.07	$-$14.92		$-$4.3540	5	0.2000
P04	5.26	6.66	$-$8.1	0.22	$-$9.63	$-$8.88	$-$2.4117	6	0.1667
P05	$-$12.18	$-$7.65	$-$20.56	$-$4.13	$-$3.84	$-$11.58	$-$9.9900	6	0.1667
P06	15.52	$-$21.37	$-$16.96	$-$13.34	$-$13.12		$-$9.8540	5	0.2000
P07	$-$26.51	$-$30.79	$-$34.1	$-$11.86	$-$16.18	$-$11.73	$-$21.8617	6	0.1667
Mean	$-$8.4874						$-$7.8413	4.8571	0.2357
Variance	127.0444						52.7442	2.1429	0.0145

**Real Data Example of Theorem** 2. Now consider the entire sample of the data from 150 participants with one or more scores; the computations are similar. Parameter $\sigma ^{2}$ was estimated as the sample variance of the whole data set, regardless of the subject and the trial. $\textrm{Var}(X_{+})$ was estimated as the sample variance of $X_{+}$, and $\mathbb {E}\left( N^{-1} \right)$ as the sample mean of 1/*N*. The data of all subjects with $N>0$ were used in all these estimates. The estimates are reported in Table 4. In conclusion, the reliability of the total score is estimated at 0.559.

**Table 4:** Estimates Needed for Computing the Unconditional Reliability

Parameter	Estimate
$\sigma ^{2}$	183.365
$Var(X_{+})$	49.179
$\mathbb {E}\left( N^{-1} \right)$	0.1391
$Rel\left( X_{+} \right)$	0.559

As Theorem 2 is based on the assumption of parallel items, one would need to check this assumption. Supplementary Material B illustrates some visual inspections that may be relevant to this. But note that if the items are parallel, the estimates based on Theorem 1 and 2 estimate the same parameter, provided that they are computed on the same data. When we applied Theorem 1 in Table 2, we used a subset of the data, and this yielded the estimate 0.663. If we use the same subset of data to estimate the reliability with Theorem 2, we obtain 0.614; the difference between the two estimates is 0.049. The size of the difference may be viewed as an indication of the extent to which the assumption of parallel items is violated. Simulations of parallel items with normally distributed true scores and error scores and reliability 0.1887 (needed to reproduce the length-stratified alpha of 0.663) suggest that this difference falls between the 98$^{\textrm{th}}$ and the 99$^{\textrm{th}}$ percentile of the sampling distribution. Although the difference between the two estimates seems significant, indicating a violation of the assumption of parallel items, the effect of the violation on the reliability estimate is modest.

A reason why the reliability is relatively low is that a small value of *N* has a large effect on $\mathbb {E}\left( N^{-1} \right)$. Therefore, if the reliability is small, we recommend to revise the data collection such that each subject has a certain minimum number of valid scores. As an example, in a Flanker Task one could consider decreasing the allotted time for answering, which would increase the number of errors.

**Comparison of Various Methods With Real Data. **One may be interested in a comparison of our outcomes with the outcomes of preexisting methods if they are applied to the ERN data. We consider (1) various versions of coefficient alpha, (2) the split-half reliabilities, and (3) variance components. For a fair comparison, we use only the data with $N\ge 2$. Recall that our first method, length-stratified alpha, yielded 0.663 as a lower bound and utilized 83% of the data, and our second method, assuming parallel items, yielded the estimate 0.559 based on 100% of the data.

1. We computed coefficient alpha for the first eight items with all participants that have eight or more items. The outcome was 0.487, based on 117 participants, so that computations use 117 $\times$ 8 / 3011 $=$ 31% of the data. The arithmetic mean of the number of observations (confined to $N\ge 2)$ was 20.99, and when we computed coefficient alpha for the first 21 items with all participants that have 21 or more items, the outcome was 0.695, based on 56 participants, which utilized 56 $\times$ 21 / 3011 $=$ 39% of the data. In view of the fact that Corollary 2 uses the harmonic mean, we repeat this computation with the harmonic mean. The harmonic mean of the number of observations (confined to $N\ge 2)$ was 10.3172, and when we computed coefficient alpha for the first 10 items with all participants that have 10 or more items, the outcome was 0.596, based on 106 participants, which utilized 106 $\times$ 10 / 3011 $=$ 35% of the data. Our lower bound 0.663, based on Corollary 1, has the advantage that it also uses data from subjects with fewer than 8, 10, or 21 observations.
2. The correlation between the mean of the first half and the mean of the second half of the scores was 0.496, yielding a split-half reliability of 0.663. If the halves were randomly selected, the mean split-half reliability over 1000 independent draws was 0.665 with a standard deviation of 0.039. This computation utilizes 100% of the data, which is therefore not entirely comparable with length-stratified alpha, which used 83% of the data. If the same 83% of the data is used to compute the split-half reliabilities, after 1000 draws the split-half reliabilities had a mean of 0.671 with a standard deviation of 0.024
3. In a variance components model, the restricted maximum likelihood estimates for the variance components of participants, items, and interaction $+$ error were 30.575, 0.138, and 151.965. Clayson et al. (2021, p. 183) recommended computing the stepped-up coefficient with the arithmetic mean, but our analysis shows that the harmonic mean should be used (a further explanation on this is found after Eq. 4). Using the arithmetic mean of the number of observations (21), the estimated reliability is $30.575/(30.575+151.965/21)=0.809.$ Using the harmonic mean (10), the estimated reliability is $30.575/(30.575+151.965/10)=0.668.$

### Correlation with a Second Test Administration

For a fixed number of items across participants, the CTT reliability of the test score $X_{+}$ equals the correlation of the test with a parallel test. The idea is that if one could replicate the test administration under similar circumstances, the reliability tells us what the correlation between the first and second test is (in the context of this article, the terms ’test’ and ’test administration’ are used interchangeably). Although in practice parallel tests are (nearly) impossible to obtain, we consider the theoretical issue of what happens to this result if the number of items is allowed to vary across participants, typical of ERPs obtained using the Flanker test. We will show that if the items within the first test are not parallel, even if the items of the second test are one-by-one parallel with the items of the first test if both items are administered, a change in the number of items in the second test, in comparison with the first test, will entail that subjects can have a different true score $T_{+}$ on the second test. Thus, even if the items of the two tests are one-by-one parallel, the test scores would not be parallel, and therefore, there is no reason to expect that the correlation of the two test scores is equal to their reliability. We study this next in more detail. In doing this, we assume in the mathematical development that the series of items in both tests are infinitely long irrespective of whether they have really been observed.

Let the items of the second test be denoted by ${X'}_{i}$, ${T'}_{i}$, and ${E'}_{i}$ and the number of items of the second test by $N'$. We assume for all $i,j\in \mathbb {N}$,

A8 $$\begin{aligned}{} & {} X'_{i} = T'_{i} + E'_{i} \end{aligned}$$

A9 $$\begin{aligned}{} & {} {\textrm{Cov}}(E'_{i},\,T'_{j}) = 0 \end{aligned}$$

We use the following assumptions. First, the items of the two tests are one-by-one parallel, that is, for all items $i\in \mathbb {N}$,

A10 $$\begin{aligned}{} & {} T_{i} = T'_{i}; \end{aligned}$$

A11 $$\begin{aligned}{} & {} {\textrm{Var}}(E_{i}) = {\textrm{Var}}(E'_{i}); \end{aligned}$$

A12 $$\begin{aligned}{} & {} {\textrm{Cov}}(E_{i},E'_{i}\,). = 0 \end{aligned}$$

Note that the definition requires A8–A10 for all $i\in \mathbb {N}$, even though only *N* items are observed in the first test, and only $N^{'}$ in the second test, where $N\ne N^{'}$ in general. The assumptions state that the equalities hold *if* the variables involved are observed, but they do not imply that all of these variables are indeed observed. This circumstance is comparable to the setup in mathematical statistics where we use an infinite sequence of random variables to obtain the central limit theorem, even though any real sample will include only a finite number of these random variables.

We do not need to assume that the errors within a test administration are uncorrelated, but we will assume that the error correlations are the same in both test administrations: for all $i,j\in \mathbb {N}$,

A13 $$\begin{aligned} {\textrm{Cov}}\left( E_{i},\,{E}_{j}\right) = {\textrm{Cov}}\left( E'_{i},\,E'_{j}\right) . \end{aligned}$$

Finally, we assume that

A14 $$\begin{aligned}{} & {} \left( N,N'\right) \, \text {is independent of all }\,T_{i},E_{i},T'_{i}\,\text {and}\, E'_{i}\, \text {jointly} \end{aligned}$$

A15 $$\begin{aligned}{} & {} N\, \text {and} \,N'\, \text {have the same probability distribution} \end{aligned}$$

The test scores on the second test are defined as

$$\begin{aligned} {X'}_{+}:= & {} \sum \limits _{i=1}^{N'} {X'}_{i} /N',\\ {T'}_{+}:= & {} \sum \limits _{i=1}^{N'} {T'}_{i} /N',\\ {E'}_{+}:= & {} \sum \limits _{i=1}^{N'} E^{'}_{i} /N'. \end{aligned}$$

We focus on two items from the same test to arrive at reliability based on one administration and denote the correlation between $X_{i}$ and $X_{j}$ as $\rho _{ij}$. Further, we denote the correlation between $X_{i}$ and ${X'}_{j}$ as ${\rho '}_{ij}$. If the items are one-by-one parallel, then we have

$$\begin{aligned} \rho _{ij}= & {} {\rho '}_{ij}\,\,\,\,\,\,\,(i\ne j)\\ \textrm{Var}\left( X_{i} \right)= & {} \textrm{Var}\left( {X'}_{i} \right) \end{aligned}$$

and ${\rho '}_{ii}$ is the reliability of $X_{i}$. The average covariance between the first *n* items of the first test and the first *m* items of the second test is

$$\begin{aligned} \bar{C}_{nm}:=\frac{1}{nm}\sum \limits _{i=1}^n \sum \limits _{j=1}^m{{\rho '}_{ij}\sqrt{\textrm{Var}\left( X_{i} \right) \textrm{Var}({X'}_{j})} }. \end{aligned}$$

To express this in terms of only parameters of the first test administration, write $\rho _{ij}^{*}:=\rho _{ij}$ if $i\ne j$ and $\rho _{ii}^{*}:={\rho '}_{ii}$, and let

$$\begin{aligned} \bar{C}_{nm}^{*}:=\frac{1}{nm}\sum \limits _{i=1}^n \sum \limits _{j=1}^m {\rho _{ij}^{*}\sqrt{\textrm{Var}\left( X_{i} \right) \textrm{Var}(X_{j})} }. \end{aligned}$$

If the items are one-by-one parallel, then $\bar{C}_{nm}^{*}=\bar{C}_{nm}$, but the point of $\bar{C}_{nm}^{*}$ is that it is entirely defined with parameters of the first test. Let

$$\begin{aligned} \pi _{nm}=P(N=n,N^{'}=m). \end{aligned}$$

We are now able to formulate Theorem 3, which allows us to express the correlation between the two test scores as a function of the parameters of the first test and the joint distribution of the test lengths ($\pi _{nm})$. The involved parameters include the item reliabilities, and we do not offer a method to estimate them, but this is irrelevant to the conclusion that we will draw from this theorem.

#### Theorem 3

Assume A1, A2, and A8–A15. The correlation between $X_{+}$ and ${X'}_{+}$ is

$$\begin{aligned} \textrm{Cor}\left( X_{+},{X'}_{+} \right) =\frac{1}{\textrm{Var}(X_{+})}\sum \limits _{n=1}^\infty \sum \limits _{m=1}^\infty {\mathrm {\,}\bar{C}_{nm}^{*}\pi _{nm}}. \end{aligned}$$

#### Proof

By the law of total covariance,

$$\begin{aligned} \textrm{Cov}\left( X_{+},X^{'}_{+} \right) =\,\mathbb {E}\left( \textrm{Cov}\left( {X_{+},X^{'}_{+}} \vert \,{N,N'}\right) \right) +\textrm{Cov}\left( \mathbb {E}\left( X_{+} \vert \,{N,N^{'}}\right) , \mathbb {E}\left( {X'}_{+} \vert \,{N,N^{'}}\right) \,\right) . \end{aligned}$$

Because $(N,N')$ is independent of $X_{+}$ (a consequence of A14), $\mathbb {E}\left( X_{+} \vert \,{N,N^{'}}\right) =\mathbb {E}(X_{+})$, and therefore, $\textrm{Cov}\left( \mathbb {E}\left( X_{+} \vert \,{N,N^{'}}\right) ,\mathbb {E}\left( X^{'}_{+} \vert \,{N,N^{'}}\right) \,\right) =0$. Therefore,

$$\begin{aligned} \textrm{Cov}\left( X_{+},X^{'}_{+} \right) =\,\mathbb {E}\left( \textrm{Cov}\left( {X_{+},X^{'}_{+}} \vert \,{N,N'}\right) \right) . \end{aligned}$$

Now

$$\begin{aligned} \textrm{Cov}\left( {X_{+},X^{'}_{+}} \vert \,{N,N'}\right) =\frac{1}{NN'}\sum \limits _{i=1}^N \sum \limits _{j=1}^{N'} {\textrm{Cov}\left( {X_{i},X^{'}_{j}} \vert \,{N,N'}\right) }. \end{aligned}$$

Because $(N,N')$ is independent of $(X_{i},X^{'}_{j})$, $\textrm{Cov}\left( {X_{i},X^{'}_{j}} \vert \,{N,N'}\right) =\textrm{Cov}\left( X_{i},X^{'}_{j} \right)$. If $i\ne j$, then $\textrm{Cov}\left( X_{i},X^{'}_{j} \right) =\textrm{Cov}\left( X_{i},X_{j} \right)$ because parallel tests have similar error correlations. If $i=j$, then $\textrm{Cov}\left( X_{i},X^{'}_{j} \right) =\mathrm {\,}{\rho '}_{ii}\textrm{Var}(X_{i})$, because $X_{i}$ and ${X'}_{i}$ are parallel. Notice that due to parallelism, $\textrm{Var}\left( X_{i} \right) =\textrm{Var}({X'}_{i})$. In sum, we have that $\textrm{Cov}\left( {X_{i},X^{'}_{j}} \vert \,{N,N'}\right) =\rho _{ij}^{*}\sqrt{\textrm{Var}\left( X_{i} \right) \textrm{Var}(X_{j})}$ for all $i,j\in \mathbb {N}$. Therefore, $\textrm{Cov}\left( {X_{+},X^{'}_{+}} \vert \,{N,N'}\right) =\bar{C}_{NN'}^{*}$, yielding

$$\begin{aligned} \textrm{Cov}\left( X_{+},X^{'}_{+} \right) =\mathbb {E}\left( \bar{C}_{NN'}^{*} \right) =\sum \limits _{n=1}^\infty \sum \limits _{m=1}^\infty {\bar{C}_{nm}^{*}\pi _{nm}}. \end{aligned}$$

Finally, because the two tests have one-by-one parallel items and *N* and $N'$ have the same probability distribution, $\textrm{Var}\left( X_{+} \right) =\textrm{Var}({X'}_{+})$. $\square$

Note that the correlation between *N* and $N'$ will affect the $\pi _{nm}$, and if the items within a test are not parallel, this will generally affect the outcome. The correlation between *N* and $N'$ will not affect $Rel(X_{+})$ as defined earlier, however, and therefore, we conclude from this theorem that *the correlation between the total scores of two tests with variable lengths will generally not be equal to the reliability of the total score, even if the items of the two tests are one-by-one parallel and the two tests have identical distributions of test lengths. *One way to understand this result is that the variable test length acts as a source of variation that is not included in the definition of reliability. We will analyze this situation in the next section using GT.

If the items *in a test* are parallel, the situation simplifies considerably. In that case $\bar{C}_{nm}=\rho \sigma ^{2}$, so that ${\sum }_{n=1}^\infty {\sum }_{m=1}^\infty {\bar{C}_{nm}\pi _{nm}} =\rho \sigma ^{2}$, regardless of the distribution $\pi _{nm}$, hence regardless of the correlation between *N* and $N'$. Furthermore, we saw earlier that $\textrm{Var}\left( X_{+} \right) =\rho \sigma ^{2}+\mathbb {E}\left( N^{-1} \right) (1-\rho )\sigma ^{2}$, which implies that we do not even need that *N* and $N'$ have the same distribution; it is sufficient that $\mathbb {E}\left( 1/N \right) =\mathbb {E}\left( 1/N' \right)$.

#### Corollary 3

Assume A1, A2 (basic CTT), A6, A7 (the items of the first test are parallel) and A8–A14 (the items of the second test are one by one parallel with the items of the first test, with similar error correlations, and the true and error score variables are independent of $(N,N^{'}))$. If $\mathbb {E}\left( 1/N \right) =\mathbb {E}\left( 1/N' \right)$, then $\textrm{Cor}\left( X_{+},{X'}_{+} \right) =\textrm{Rel}(X_{+})$.

#### Integration of Reliabilities of Different Stimulus Types

This section briefly discusses how the above methods can be applied if the ERPs are obtained from different stimulus types, such as ‘SSSSS’, ‘SSHSS’, ‘HHSHH’, and ‘HHHHH’ in the Flanker Task. In such cases, one may consider it implausible that ERPs from different stimulus types are parallel. Nevertheless, the method of Corollary 1 can still be used because it does not require parallel items. If the items within each stimulus type are parallel, while items from different stimulus types are not parallel, a better estimate can be obtained with the following method: (1) estimate the reliabilities within each stimulus type using the methods of Theorem 2 and (2) integrate the ensuing reliabilities with the formula for stratified reliability of composite tests, $1-{\sum }_{g=1}^G {\sigma _{g}^{2}\left( 1-\rho _{g} \right) } /\,\textrm{Var}(X_{+})$, discussed after Theorem 1.

## Comparison with Generalizability Theory Approaches

Several authors have adopted the use of GT for ERP scores. Baldwin et al. (2015) and Clayson and Miller (2017a; b) described a model with persons and trials as random factors. They included diagnostic category as a fixed factor, where persons are nested within diagnostic categories such as anxiety disorder and major depressive disorder. The authors estimated the generalizability coefficients in each diagnostic category separately, so for the present discussion it suffices to consider only one diagnostic category and thus omit diagnostic category as a factor. In addition, Clayson et al. (2021) described a model that includes the factors persons, trials, and occasions. Within a single diagnosis group and with data of only a single occasion, the model these authors proposed includes only persons and trials as random factors.

Because, in contrast with CTT, trial (or item) is now considered a random factor, we will slightly change the notation and write the score of a participant *p* on item *i* as *X*(*p*, *i*). The model with participant effects ($\tau _{p})$, trial effects ($\beta _{i})$, interaction effects ($\gamma _{pi})$, and a residual ($\varepsilon _{pi})$ can be written as

$$\begin{aligned} X(p,i)=\mu +\tau _{p}+\beta _{i}+\gamma _{pi}+\varepsilon _{pi}. \end{aligned}$$

Various methods exist for estimating the variance components corresponding to $\tau _{p}$, $\beta _{i}$, and $\gamma _{pi}+\varepsilon _{pi}$. Clayson et al. (2021) recommended Bayesian hierarchical models. Denote the variance components $\sigma ^{2}(\tau )$, and so on. The authors defined the *dependability* coefficient for subjects with *n* trials as

1 $$\begin{aligned} \text {Dep}\left( X_{+},n \right) =\frac{\sigma ^{2}(\tau )}{\sigma ^{2}\left( \tau \right) +\,\frac{1}{n}[\sigma ^{2}\left( \beta \right) +\sigma ^{2}\left( \gamma +\varepsilon \right) ]}. \end{aligned}$$

Baldwin et al. (2015, p. 792) assumed furthermore that $\sigma ^{2}\left( \beta \right) =\sigma ^{2}\left( \gamma \right) =0$, leading to the special case

2 $$\begin{aligned} \text {Dep}\left( X_{+},n \right) =\frac{\sigma ^{2}(\tau )}{\sigma ^{2}\left( \tau \right) +\,\frac{1}{n}\sigma ^{2}\left( \varepsilon \right) }. \end{aligned}$$

Writing $\rho =\sigma ^{2}(\tau )/(\sigma ^{2}\left( \tau \right) +\sigma ^{2}(\varepsilon ))$, we can rewrite the coefficient of Baldwin et al. as

3 $$\begin{aligned} \text {Dep}\left( X_{+},n \right) =\frac{n\rho }{1+(n-1)\rho }. \end{aligned}$$

To compare this result with our own results, we note that if we define the true scores as $T_{i}\left( p \right) =\mu +\tau _{p}+\beta _{i}+\gamma _{pi}$, the assumption $\sigma ^{2}\left( \beta \right) =\sigma ^{2}\left( \gamma \right) =0$ implies that the items are tau-equivalent, i.e., $T_{i}=T_{j}$ for all $i,j\in \mathbb {N}$. Baldwin et al. (2015) used the same value of $\sigma ^{2}\left( \varepsilon \right)$ regardless of the included items or participants, so they treat the items as if they are parallel. In Corollary 2, we concluded for the situation of parallel items that, with $H=1/\,\mathbb {E}\left( N^{-1} \right)$ (the harmonic mean of *N*),

4 $$\begin{aligned} \text {Rel}\left( X_{+} \right) =\frac{H\rho }{1+(H-1)\rho }. \end{aligned}$$

We will now discuss the differences between the approach of Baldwin et al. (2015) and our own analysis. The most obvious difference is that Baldwin et al. use Equation (3), which uses a fixed number of trials *n*, whereas we use Equation (4), which uses the harmonic mean *H* of a variable number of trials. Baldwin et al. thus compute *conditional* dependability coefficients, given a value of *n*, but they do not discuss how these conditional coefficients can be integrated into a single *unconditional* coefficient that summarizes the reliability or dependability in a population of persons having different values of *n*. Clayson et al. (2021, p. 183) recommend integration by using a formula that is equivalent to (3) and replace *n* by the arithmetic mean or median of *N*, but this seems to be an ad hoc formula without proof of correctness. Our analysis shows that this integration can be done with essentially the same formula, which is the Spearman–Brown formula, replacing the fixed test length with the harmonic mean of the test lengths. Our formula has the advantage that it is mathematically proven to produce the unconditional reliability when this is defined in the conventional manner as the true score variance divided by the observed score variance. The harmonic mean ($H=1/{\,}\mathbb {E}\left( N^{-1} \right) )$, rather than the arithmetic mean (denoted here with $A=\mathbb {E}\left( N \right) )$, is used because the overall error variance is the expected value of the individual error variances $\mathrm {\,}\frac{1}{n}\sigma ^{2}\left( \varepsilon \right)$, which is $\sigma ^{2}\left( \varepsilon \right) /H$ and not $\sigma ^{2}\left( \varepsilon \right) /A$. In general, $H<\,A$ if $N>0$ and $\textrm{Var}\left( N \right) >0$, so using the arithmetic mean produces estimates that are too optimistic. Mathematically, Equation (4) is more general than Equation (3), because the latter can be viewed as a special case of the former when the test length is fixed. The two formulas can be complementary in their applications. Equation (3) can be useful in clinical settings if, after the test administration, one wants to decide whether enough trials have been observed for a given patient with known *n*, even if the estimate of $\rho$ is based on data with many patients with variable *N*. Equation (4) can be used in research where a single reliability value is needed for a group of persons with variable *N*.

The second difference is the method for estimating $\rho$. Baldwin et al. (2015) advocate the use of a Bayesian hierarchical model to estimate the variance components and their ratio. In Corollary 2a, we concluded that it suffices to estimate $\sigma ^{2}$ (the variance of all scores) and $\textrm{Var}\left( X_{+} \right)$ and $\mathbb {E}\left( N^{-1} \right)$. These are simply variances and means of observed variables, and in the two examples of Theorem 2 we demonstrated that these quantities can easily be estimated with the corresponding sample moments. Our analysis was mainly concerned with the relations between parameters, and the examples were merely given with the purpose to clarify the results, not to claim that this is the best estimation method. Any estimate of $\rho$ may be inserted in Corollary 2b (the Spearman–Brown formula with harmonic mean of *N*) to obtain an estimate of the overall reliability. We discuss the merits of the method of Baldwin et al. and compare them with our estimation method based on Corollary 2a.

According to Baldwin et al. (2015), the advantages of their method are that it does not produce negative variance component estimates and that “computing interval estimates and hypothesis tests for variance components and dependability coefficients is straightforward” (ibid., p. 794). Our method is based on the sample variances of $\sigma ^{2}$ and $\textrm{Var}\left( X_{+} \right)$, which cannot be negative either. Note that the method of Baldwin et al. assumes normal distributions for the components, whereas our method does not require any distributional assumptions whatsoever. The method of Baldwin et al. produces interval estimates, but in doing this it relies heavily on the assumption of normality. Ogasawara (2006) and Maydeu-Olivares et al. (2007) compared asymptotic distribution-free (ADF) estimators and normal theory estimators for coefficient alpha, and Maydeu-Olivares et al. concluded that “for sample sizes over 100 observations, ADF intervals are preferable, regardless of item skewness and kurtosis” (ibid., p. 157). Braschel et al. (2015) and Coffman et al. (2008) also noted lack of robustness of estimates of intraclass correlations based on normal theory, and Coffman et al. provided the ADF distribution of sample intraclass correlations. Using Bayesian methods does not render estimators invulnerable to violations of normality. Ionan et al. (2014) compared various frequentist and Bayesian methods for interval estimation of the intraclass correlation in a two-way crossed random effects model and concluded that “none of the methods work well if the number of levels of a factor are limited and data are markedly non-normal” (ibid., p. 1). This does not mean that our method is necessarily preferable, however; hypothesis testing and interval estimation of $\sigma ^{2}/\,\textrm{Var}\left( X_{+} \right)$, a ratio of two dependent variances, have similar problems if data are non-normal (Wilcox, 1990, 2015). Further research is needed to determine the optimal estimation method for small non-normal data with random numbers of observations.

A third difference is that we provide an analysis of what happens if the test administration is repeated with possibly a different number of trials. Baldwin et al. (2015) did not discuss this matter.

Clayson et al. (2021) generalized the model of Baldwin et al. (2015) to a setting with multiple occasions. Applied to a setting with a single occasion, the main difference with Baldwin et al. is that Clayson et al. do not assume $\sigma ^{2}\left( \beta \right) =\sigma ^{2}\left( \gamma \right) =0$, leading to Equation (1) instead of Equation (2). A comparison of our analysis with Clayson et al. follows roughly the same lines as our comparison with Baldwin et al. Clayson et al. describe *conditional* dependability coefficients, given a fixed number of trials, whereas our method describes how we can integrate coefficients for different numbers of trials into an *unconditional* coefficient. More specifically, if we assume that the components of $\tau ,\beta ,\gamma ,\varepsilon$ are independent of *N*, then $\textrm{Var}\left( X_{+} \right) = \quad \mathbb {E}\left( \textrm{Var}\left( X_{+} \vert \,N\right) \right) ={\sum }_{n=1}^\infty {\textrm{Var}\left( X_{+} \vert \,{N=n}\right) \pi _{n}} ={\sum }_{n=1}^\infty {\{\sigma ^{2}\left( \tau \right) +\mathrm {\,}\frac{1}{n}[\sigma ^{2}\left( \beta \right) +\sigma ^{2}\left( \gamma +\varepsilon \right) ]\}\pi _{n}} =\sigma ^{2}\left( \tau \right) +\mathbb {E}\left( \frac{1}{N} \right) [\sigma ^{2}\left( \beta \right) +\sigma ^{2}\left( \gamma +\varepsilon \right) ]$. The unconditional dependability is therefore

5 $$\begin{aligned} {\textrm{Dep}}(X_{+}) =\frac{\sigma ^2(\tau )}{\sigma ^{2}(\tau )+\mathbb {E}({\frac{1}{{{N}}}}) [{\sigma }^{2}({\beta })+{\sigma }^{2}({\gamma } + {\varepsilon })]}. \end{aligned}$$

Using $\rho '=\sigma ^{2}(\tau )/[\sigma ^{2}\left( \tau \right) +\sigma ^{2}\left( \beta \right) +\sigma ^{2}\left( \gamma +\varepsilon \right) ]$, we can rewrite Equations (1) and (5) as

6 $$\begin{aligned} {\textrm{Dep}}(X_{+},{n})= & {} \frac{{n\rho '}}{1 + (n- 1)\rho '}, \end{aligned}$$

7 $$\begin{aligned} {\textrm{Dep}}(X_{+})= & {} \frac{H\rho '}{1 + (H-1)\rho '}. \end{aligned}$$

Clayson et al. (2021) estimated the variance components using a Bayesian hierarchical model, but given the previous discussion, we are not convinced that this is the best estimation method. Our method to integrate various coefficients works regardless of the estimation method used for the reliability or generalizability. We have suggested coefficient alpha in Corollary 1 because it can be interpreted both in CTT and in GT (see also Sijtsma & Pfadt, 2021a; b).

## Discussion: Contributions of Our Study to the Theory and Practice of Reliability

We have extended CTT with new formulas to compute the reliability in situations where the number of items per subject is a random variable. These formulas can be applied to data of performance monitoring ERPs such as the ERN and Pe, where the number of relevant trials depends on the performance of the participant. We studied this for the Eriksen Flanker Task, but our theory can also be applied in other tasks in which ERN and Pe measurements can be obtained, such as Go / NoGo tasks and Stroop tasks (see Baldwin et al. 2015). Furthermore, we illustrated our theory with time-window mean amplitude scores, but our formulas are equally valid for other EEG scores such as peak amplitude or peak latency.

The first method we created is based on a reliability formula for a stratified sample. This method can be used in combination with existing reliability estimates such as alpha or omega, applied to each subgroup with equal test length. The limitation of this method is that it requires that each subgroup of participants with the same test length is large enough to estimate the reliability accurately. This requirement might be difficult to meet, although fortunately, for the field of psychophysiology, a trend toward the use of larger samples is observed (Kissel & Friedman, 2023). If the requirement is not met, then subgroups with different test lengths have to be combined, which leads to loss of data. The reason for this data loss is that alpha has to be computed on a rectangular data matrix; if groups with $N\mathrm {\,=\,}k$ and$\mathrm {\,}N\mathrm {\,=\,}k+ 1$ items are combined, then either alpha is computed with $k+1$ items and the participants with $N{\,=\,}k$ are discarded, or alpha is computed with *k* items and the data of the $\mathrm {(}k+1\mathrm {)}$th item are discarded. In our example, 83% of the data could be used in our example of length-stratified alpha. However, maybe it is not really necessary to estimate the reliability in each subgroup with the same accuracy as one would desire in the total group. The stratification formula combines the subgroup reliabilities in a weighted sum, and the standard error of the total reliability can be less than each of the contributing standard errors. Our first simulation of standard errors of length-stratified alpha, reported in “Example of Theorem 1,” gave promising results. Further research is needed to construct interval estimates of this version of stratified reliability and to provide sample size recommendations. The second method we proposed only requires two variance estimates and one mean to compute, which makes it very easy to apply. Moreover, our second method uses 100% of the data. Its limitation is that it requires that the items are parallel.

Our analysis shows that reliability estimation of the ERN and Pe data with CTT is very well possible. The advantage of CTT is that the greater simplicity of having only a single facet allows us to focus on an aspect that did not receive attention in the GT treatments, which is that the number of items is also a random variable. In contrast with earlier treatments of GT, we were able to define a single reliability coefficient that combines all subgroups with different numbers of items. Our analysis shows that the *harmonic mean* of the number of items, rather than the arithmetic mean, relates the variance components to the overall reliability, and this result is relevant in both CTT and GT approaches. Our analysis also clarified that even if items on a second test administration are parallel with the items of the first test administration, their total scores may not be parallel if the number of items changes between the test administrations. We generalized our approach to data that are stratified on other variables in Supplementary Material C. We pointed out that Corollary 2 and Eq. (4) (i.e., the Spearman–Brown formula with the harmonic mean of test lengths) can also be applied in designs where randomly selected raters from one population are nested within objects, with different sample sizes per object. This formula may be useful in studies of performance evaluations of health care organizations where each organization is rated by a sample of their patients, where sample sizes are usually different (e.g., Ellis, 2013; Ogasawara, 2021)—although the situation is complicated by the need for a casemix correction.

We contend that CTT still has its merits if a detailed analysis of reliability is needed. This study shows that CTT does not always require parallel items as some authors suggest and put forward as a limiting condition for using CTT (Clayson & Miller, 2017a, p. 72). The simplicity of CTT is attractive in the present context where it enables the researcher to estimate reliability in a simple way, addressing the problem of obtaining a single reliability coefficient with variable test lengths that more complex methods seem to obscure. In doing so, the present work provides a crucial and necessary contribution to advancing ERP studies of individual differences.

## Supplementary Information

Below is the link to the electronic supplementary material.

Supplementary file 1 (docx 182 KB)

## Declarations

### Data Availability Statement

The data of Tables 2 and 4 and Figures S1-S5 and the code that generated it are available in the Open Science Framework repository at 10.17605/OSF.IO/KZY3D
