# Supplementary material for: Reliability Theory for Measurements with Variable Test Length, Illustrated with ERN and Pe Collected in the Flanker Task
Source: Psychometrika. 2024 Jul 21;89(4):1280–303. doi: 10.1007/s11336-024-09982-5 (PMC11582099; doi:10.1007/s11336-024-09982-5)
Supplement: Supplementary file 1 — (docx 182 KB) [file 11336_2024_9982_MOESM1_ESM.docx]

**Supplementary Material A: Sample Descriptives, Recording and (Pre-)Processing of the EEG Data**

The sample consisted of *n* = 158 participants with a mean age of *M* = 22.22 years (*SD* = 2.56, range 17-32 years) of whom 59.5% was male and 40.5% female. Information on age was missing for one person. EEG was recorded using the Biosemi ActiveTwo system with 32 active Ag/AgCl electrodes placed by means of a head cap according to the 10–20 system, and two additional active Ag/AgCl electrodes placed at positions FCz (inclination θ= 23.0, azimuth φ = 90.0) and CPz (θ= 23.0, φ = -90.0). An electro-oculogram (EOG) was recorded with electrodes placed above and below the left eye (vertical EOG) and at the outer canthi of both eyes (horizontal EOG). Reference electrodes were placed at the left and right mastoid. Online referencing was done using the CMS electrode. All signals were digitized with a sampling rate of 512 Hz and 24-bit resolution.

The data were analyzed offline using Brain Vision Analyzer 2 software (version 2.2.0.7383). Data were first referenced to the mathematically linked mastoid electrodes. Then, 4^th^ order infinite impulse response (IIR) zero phase shift Butterworth filters were used for high-pass filtering (cut-off at 0.10 Hz), low-pass filtering (cut-off 30.00 Hz), and notch filtering (at 50.00 Hz, to filter out powerline artifacts). Data were subsequently segmented into epochs ranging from 100 ms before participants responded to 600 ms after response. Then, ocular artifact correction according to the algorithm by Gratton et al. (1983) and baseline correction (using the 100 ms pre-response period) were applied. Finally, extreme amplitudes (those below -100 µV and above 100 µV) were rejected using automatic detection. No interpolation was applied. Overall, 2.31% of (error and correct trial) segmented data was removed. The ERN was exported as the mean amplitude at electrode FCz in the 25-100 ms time window; the Pe was exported as the mean amplitude at Pz in the 200-400 ms time window. The grand averaged waveforms at those electrodes are presented in Figure S1. Markers indicating whether a correct, incorrect, or too slow response was given on a trial were also exported. R code was used to extract only the amplitudes from trials on which an incorrect response had been given.

Data and code (including Brain Vision Analyzer history templates) are available at <https://osf.io/kzy3d/?view_only=3b0fd150ed2148cb9eb2d66c55e2cfc1>

**Figure S1**

*Grand Averaged ERPs at the FCz Electrode (Left) and Pz Electrode (Right).*


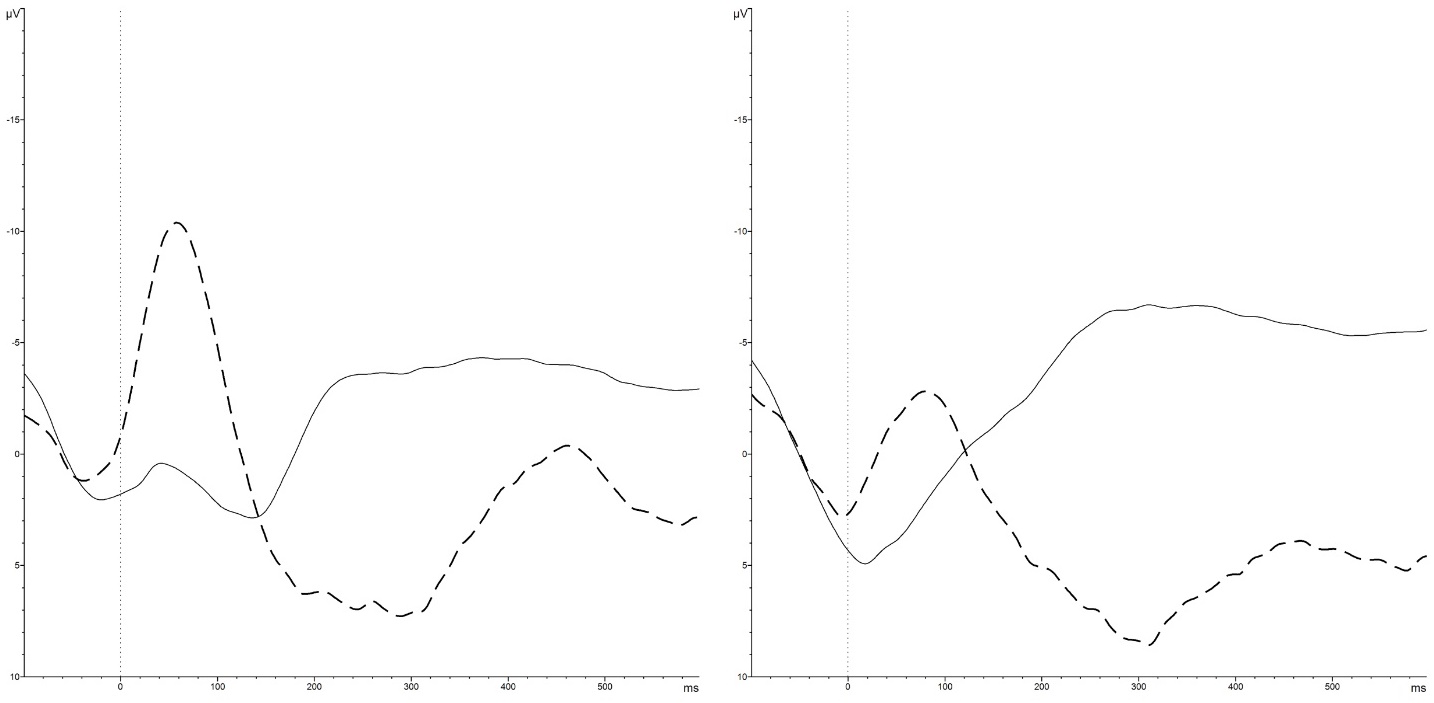


*Note.* The solid lines represent electrophysiological activity after participants provide a correct response (*n* = 158); the dashed lines represent the activity after an error (*n* = 151).

**References**

Gratton, G., Coles, M.G. & Donchin, E., (1983). A new method for off-line removal of ocular artifact. *Electroencephalography and clinical neurophysiology, 55*, 468-484. <https://doi.org/10.1016/0013-4694(83)90135-9>

**Supplementary Material B: Autocorrelations and Descriptive Statistics of ERN and Pe**

**Autocorrelations**

Within each participant, the ERPs form a time series, and an important question before further analysis commences, is to which extent the scores of subsequent error trials are uncorrelated. The scores may very well be correlated if data from multiple participants are combined, but the question here is whether they are uncorrelated *within* subjects. The reason for this question is that both CTT and GT assume that error scores are uncorrelated. In the CTT version of Lord and Novick (1968), this implies that the observed scores should be uncorrelated within subjects. In GT, too, it is assumed that subsequent responses of the same participant are sampled independently. This assumption is typically not tested in ERP data, and one of our innovations is to test it prior to further analysis, as will be discussed now.

A test of within-subject correlations can be based on autocorrelations. For a single subject with $N$ scores, the first-order (or lag 1) autocorrelation is defined as the product-moment correlation between the $i$-th score and the $(i+1)$-th score, with $i$ ranging from 1 to $N$. This can be computed within each subject with three or more observations. For example, for the first participant in Figure 1 of the main text, this would be the correlation of the paired observations $\left( -9,-16 \right), \left( -16,-10 \right), \left( -10,-5 \right)$, which is -0.219. Autocorrelations such as this are routinely studied in time series analysis. We studied first-order autocorrelations in two ERP data files, one set for ERN data and the other set for Pe data. For 138 participants out of 158 in total who produced at least three scores, we computed first-order autocorrelations. Figure S2 shows histograms of the $P$-values resulting from the Box-Ljung statistic of the first-order autocorrelations for ERN (upper panel) and Pe (lower panel). If all true autocorrelations are zero, we expect uniform histograms for the observed autocorrelations. The smallest $P$-values are .006 (ERN data) and .00117 (Pe data), and after Bonferroni-correction, i.e. multiplying each $P$-value with $N_{participants}=138$, these values were .83 and .16, respectively. In the ERN data, 13 participants (9.4%) had an uncorrected $P$-value smaller than .05 and in the Pe data, 6 participants (4.3%) had an uncorrected $P$-value smaller than .05. Note that the statistical power might be small for participants with a small number of observations, and that this can lead an almost uniform distribution even if the null hypothesis is false. Therefore, we also show the histograms of the $P$-values for the 50% subsample of participants with the highest number of observations, in Figure S3.

Our conclusion is that the evidence of non-zero autocorrelations is thin, based on lag 1 autocorrelations, and that both histograms give tentative evidence of only small deviations from uniformity. Therefore, we continue as if all autocorrelations are zero, and assume uncorrelated ERPs within participants, which justifies the use of CTT and GT formulas that require uncorrelated errors (this is not yet sufficient to assume parallel items, however).

If autocorrelations are checked in other data, we suggest to use detrended scores, such as the difference between subsequent scores, rather than the raw scores. The reason for this is that a trend in the means, such as gradually decreasing means, can cause a non-zero autocorrelation even if the measurement errors are uncorrelated. If it is found that the detrended scores have non-zero autocorrelations, use of either CTT would be questionable. Use of GT might still be possible if one uses a multilevel model with correlated errors.

**Figure S2**

*Histograms for* $P$*-values of 138 Participants Each Producing at Least 3 ERN or Pe Scores.*


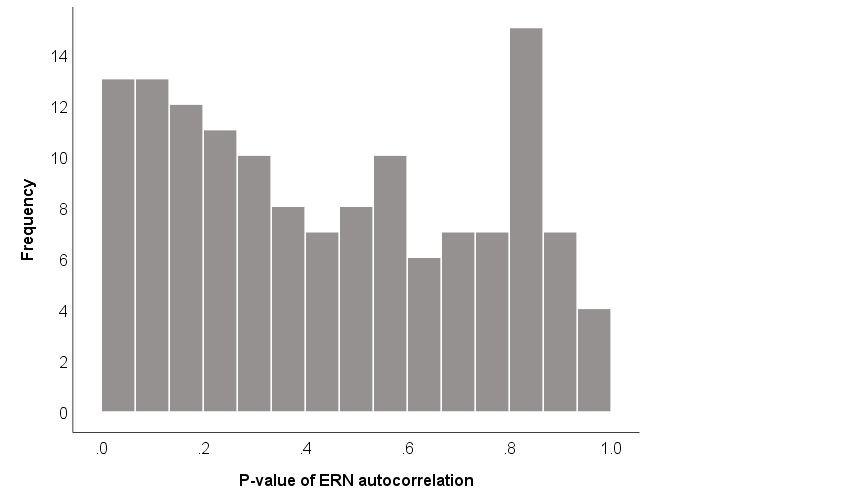


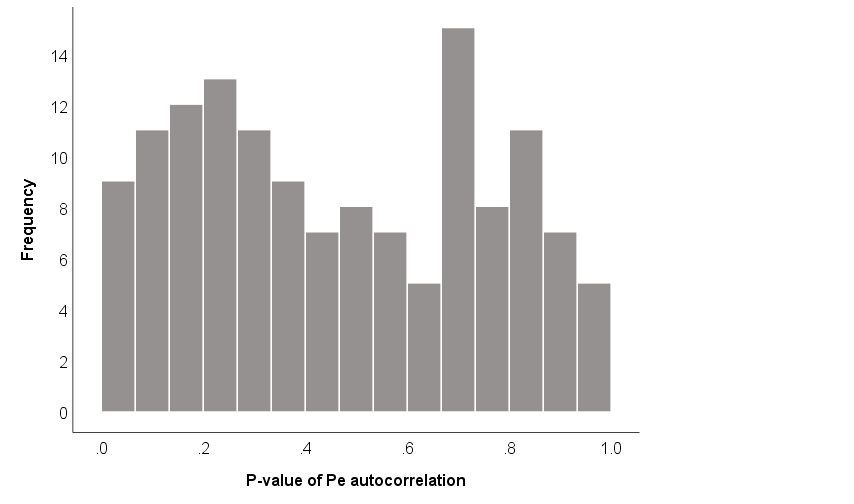


**Figure S3**

*Histograms for* $P$*-values of Participants With 17 or More Observations.*


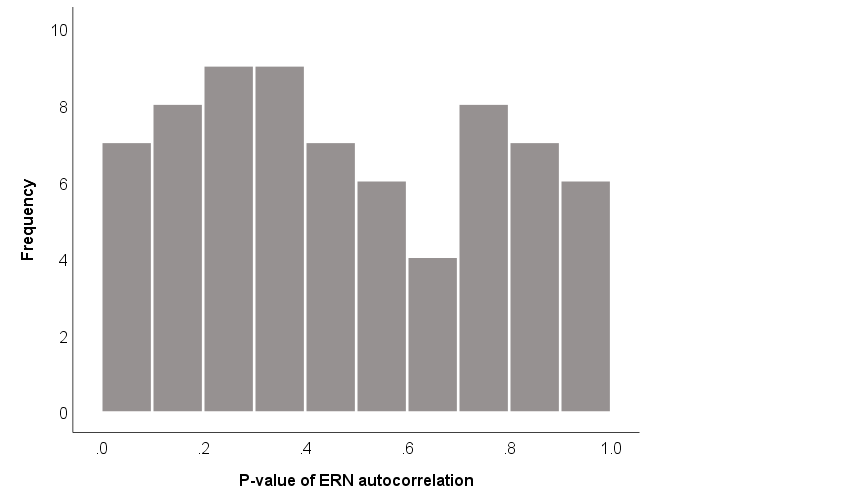


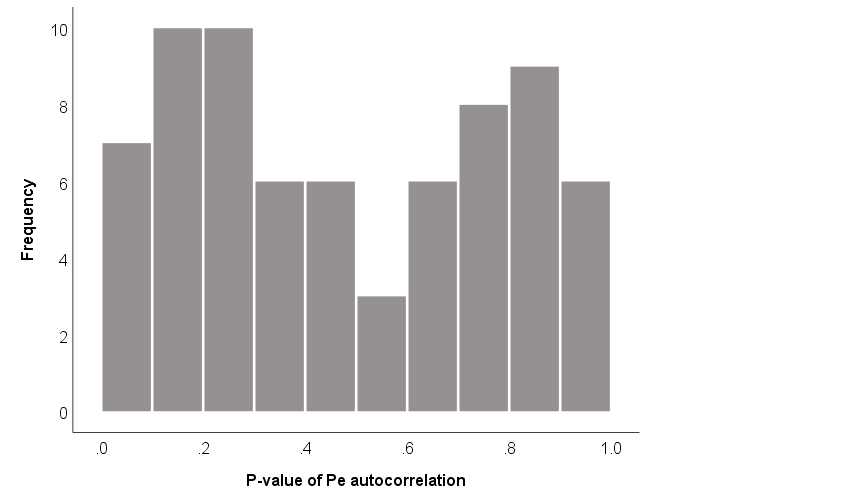


**Descriptive Statistics**

Figure S4 shows the development of mean and standard deviation in ERN over trials, based on the condensed data matrix. Figure S5 shows the same for Pe. Both figures also display the square root of the number of observation per trial. The square root is taken in order to scale the three quantities conveniently in the same plot. Note that the number observations per trial decreases consistently. This is a logical consequence of the fact that the condensed data matrix is used. The number of participants with at least $k$ observations is decreasing with $k$. For example, there were 106 participants with 10 or more error trials, and the mean and standard deviation of the ERN of these participants are displayed above error trial 10. Moving from trial 10 to trial 11, we lose the seven participants who had 10 observations but nut 11 observations. Thus, the counts per trial can only decrease or stay equal; they cannot increase.

A consequence of the fact that the numbers of observations decrease from left to right in Figure S4 and Figure S5, is that the means and standard deviation become less precise estimates for their population values. Therefore, we displayed them only for sample sizes of at least 20 participants, which is only the first 36 error trials. Taking this into account, we conclude that there is no substantial change in mean or standard deviation over error trials; that is, there is no obvious evidence of a practice effect in the ERN and Pe data. Statistical tests confirmed this.

A related question is whether the error trials can be considered as parallel items. This assumption would be contradicted if the mean or variance changed over error trials within the same group of participants. The above plot does not answer this question, since the participant group changes from left to right in the plot. A proper analysis can be based on a multilevel analysis for repeated measures, as discussed by Clayson and Miller (2017a,b). However, we do not have a methodological innovation on this point, and therefore we will not conduct this analysis.

**Figure S4**

*Trend of Mean and SD in ERN Data, Based on Condensed Data Matrix for First 36 Trials*

**
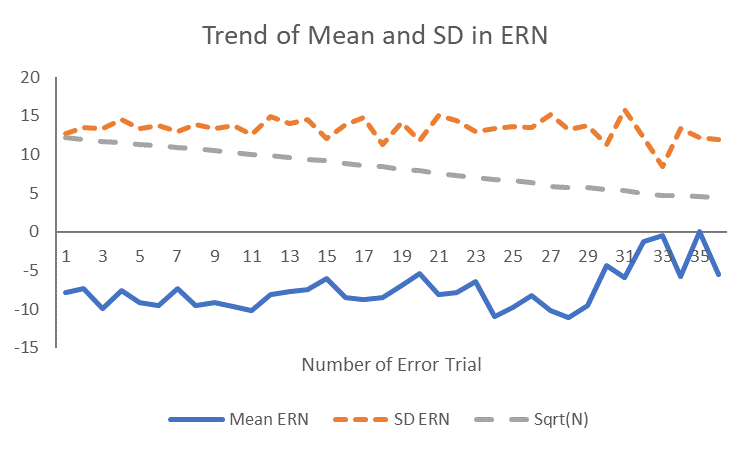
**

**Figure S5**

*Trend of Mean and SD in Pe Data, Based on Condensed Data Matrix for First 36 Trials*

**
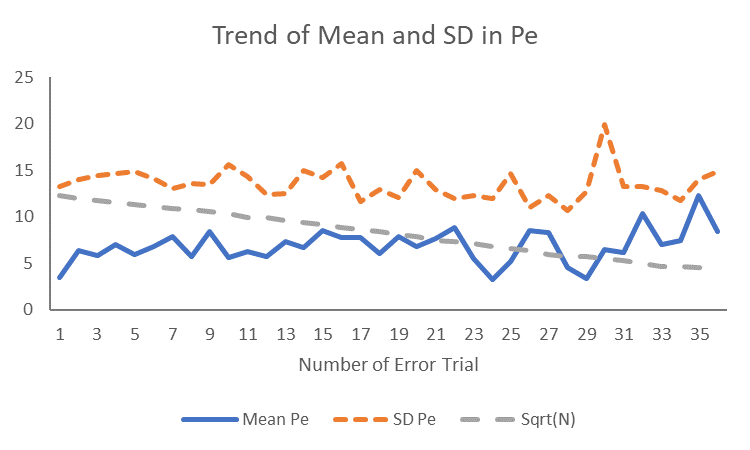
**

**Supplementary Material C: Generalization. Combining Reliabilities of Different Subpopulations**

**and Unequal Test Lengths**

The equalities that lead to Theorem 1 are also true if $N$ is replaced by another discrete variable. This leads to the following generalization. In addition to the assumptions set out earlier, we assume that there is a discrete variable $M$ with outcomes in $\mathbb{N}$, and for all $i,j,n, m\mathbb{\in N}$,

|  | $\mathbb{E}\left( E_{i} \vert N=n,M=m \right)=0$ | A16 |
| --- | --- | --- |
|  | $\mathrm{Cov}\left( E_{i}, T_{j}\vert N=n,M=m \right)=0$ | A17 |

For an arbitrary function $f\mathbb{:N\times N\to R}$ we define the total scores as

$$X_{f}=\sum_{i=1}^{N} X_{i}f(N,M)$$

$$T_{f}=\sum_{i=1}^{N} T_{i}f(N,M)$$

$$E_{f}=\sum_{i=1}^{N} E_{i}f(N,M)$$

An important example of this is $M=1$ and $f\left( N,M \right)=1/N$; then $X_{f}=\frac{\sum_{i=1}^{N} X_{i}}{N}=X_{+}$. This is the case treated in Theorem 1. Another important example is that $M$ is some discrete grouping variable, e.g. ‘nationality’, and that $N=n$ is fixed, and $f\left( N,M \right)=1$. This is a case where the sum score over the same items is used in different subpopulations. Combinations of these two examples are allowed too.

We can now repeat the reasoning that lead to Theorem 1 almost word by word, where conditioning on $N$ is replaced by conditioning on $(N,M)$:

**Lemma 2**. Assume A1, A2, A16, A17. Then

$\mathrm{Cov}\left( E_{f}, T_{f} \right)=0$.

*Proof*. By the law of total covariance, we have

$$\mathrm{Cov}\left( E_{f}, T_{f} \right)\mathbb{=E(}\mathrm{Cov}\left( E_{f}, T_{f}|N,M \right))+\mathrm{Cov}\mathbb{(E(}E_{f}|N,M),\mathbb{E(}T_{f}|N,M))$$

In the first term, we have

$$\mathrm{Cov}\left( E_{f}, T_{f}|N=n, M=m \right)=\sum_{i=1}^{n} \sum_{j=1}^{n} \mathrm{Cov}\left( E_{i}, T_{j}|N=n,M=m \right){f(N,M)}^{2}=0$$

Therefore, $\mathbb{E}\left( \mathrm{Cov}\left( E_{f}, T_{f}|N,M \right) \right)=0.$

In the second term, we have

$$\mathbb{E}\left( E_{f} | N=n,M=m \right)\mathbb{=E}\left( \sum_{i=1}^{N} E_{i}f\left( N,M \right) | N=n,M=m \right)$$

$$=\sum_{i=1}^{n} f(N,M)\mathbb{E}\left( E_{i} | N=n,M=m \right)=0$$

Therefore, $\mathrm{Cov}\left( \mathbb{E}\left( E_{f} | N,M \right),\mathbb{E}\left( T_{f} | N,M \right) \right)=0$.

Q.E.D.

From the lemma it follows immediately that $\mathrm{Var}\left( X_{f} \right)=\mathrm{Var}\left( T_{f} \right)+\mathrm{Var}\left( E_{f} \right)$.

Define the *unconditional reliability* of the total score as

$$\mathrm{Rel}\left( X_{f} \right):=\frac{\mathrm{Var}(T_{f})}{\mathrm{Var}(X_{f})}$$

Let us now consider some parameters that can be defined after stratifying the subject population on the basis of $(N,M)$. The *conditional reliability* of the total score is now defined as

$$\rho_{nm}:=\frac{\mathrm{Var}(T_{f}|N=n,M=m)}{\mathrm{Var}(X_{f}|N=n,M=m)}$$

Furthermore, let us write the conditional observed variance as

$$\sigma_{nm}^{2}:=\mathrm{Var}(X_{f}|N=n,M=m)$$

and the fraction of the subjects with this number of observations as

$$\pi_{nm}:=P(N=n,M=m)$$

**Theorem 4.** Assume A1, A2, A16, A17. The unconditional reliability of the total score $X_{f}$ is given by

$$\mathrm{Rel}\left( X_{f} \right)=1-\frac{\sum_{m=1}^{\infty} \sum_{n=1}^{\infty} (1-\rho_{nm})\sigma_{nm}^{2}\pi_{nm}}{\mathrm{Var}(X_{f})}$$

*Proof*. Since $\mathrm{Var}\left( X_{f} \right)=\mathrm{Var}\left( T_{f} \right)+\mathrm{Var}\left( E_{f} \right)$, the definition of unconditional reliability implies

$$\mathrm{Rel}\left( X_{f} \right)=1-\frac{\mathrm{Var}\left( E_{f} \right)}{\mathrm{Var}(X_{f})}$$

By the law of total variance, $\mathrm{Var}\left( E_{f} \right)\mathbb{=E}\left( \mathrm{Var}\left( E_{f} | N,M \right) \right)+\mathrm{Var}\mathbb{(E}\left( E_{f} | N,M \right))$. By A16, $\mathbb{E}\left( E_{f} | N=n,M=m \right)=0$ and therefore $\mathrm{Var}\left( \mathbb{E}\left( E_{f} | N,M \right) \right)=0$, so

$$\mathrm{Var}\left( E_{f} \right)\mathbb{=E}\left( \mathrm{Var}\left( E_{f} | N,M \right) \right)$$

$$=\sum_{m=1}^{\infty} \sum_{n=1}^{\infty} \mathrm{Var}\left( E_{f} | N=n,M=m \right)\pi_{nm}$$

$$=\sum_{m=1}^{\infty} \sum_{n=1}^{\infty} (1-\rho_{nm})\sigma_{nm}^{2}\pi_{nm}$$

Q.E.D.

Consider the case where $N$ is a function of $M$, and $f\left( N,M \right)=1/N$. This means that $X_{f}=X_{+}$ is used with the same items in each subpopulation that is defined by $M$. Denote the reliability, variance, and probability of subpopulation $[M=m]$ by $\rho_{m}$, $\sigma_{m}^{2}$, and $\pi_{m}$, respectively.

**Corollary 4.** Assume A1, A2, A16, A17, and let $N$ be a function of $M$, and $f\left( N,M \right)=1/N$. The unconditional reliability of the total score $X_{f}$ is given by

$$\mathrm{Rel}\left( X_{+} \right)=1-\frac{\sum_{m=1}^{\infty} (1-\rho_{m})\sigma_{m}^{2}\pi_{m}}{\mathrm{Var}(X_{+})}$$

Therefore, the formula of Theorem 1, where $N$ was supposed to be the number of items, is also valid if $N$ is any other discrete variable while the number of items is fixed. An interesting example is that organizations are rated by persons, where each organization is rated by a different sample of persons, and where the sample sizes may be different. For example, hospitals can be rated by patients, or schools can be rated by students. In this case the persons have the same role in the intraclass correlation as items have in coefficient alpha. If the persons are randomly selected from the same population, in a random order with replacement, we may consider the first measurement and the n-th measurement as parallel, even though two fixed persons are probably not parallel. In this case, if $M$ indicates the organization, $N$ is a function of $M$, and if we measure each organization with its mean rating then we use $f\left( N,M \right)=1/N$. Using Corrolary 4, and similar reasoning as in Corrolary 2, we obtain that with $H=1/ \mathbb{E}\left( N^{-1} \right)$, if $\rho$ is the intraclass correlation of a single measurement, the aggregate intraclass correlation of $X_{+}$ with random numbers of measurements is given by $\frac{H\rho}{1+(H-1)\rho}$. However, in practical applications one can typically not assume that patients of different hospitals, or students of different schools, are drawn from the same population, and then a casemix correction is needed, which complicates matters.
